# Liberal transfusion strategies reduce sepsis risk and improve neurological recovery in acute brain injury: an updated systematic review and meta-analysis

**DOI:** 10.1186/s13054-025-05397-5

**Published:** 2025-05-06

**Authors:** Nhan Nguyen, Vinh Quang Tri Ho, David Downes, Bao Nghi Tran, Vy Ngoc Dan Nguyen, Emmanuel Mark M. Velasco

**Affiliations:** 1 Faculty of Medicine, University of Debrecen, 1 Egyetem ter, Debrecen, 4032 Hungary; 2 Department of Anesthesiology and Pain Medicine, Toronto General Hospital, Toronto, Canada; 3 Faculty of Medicine, Dentistry and Health Sciences, Department of Nursing, The University of Melbourne, Melbourne, Australia; 4 Department of Rural Medicine, The University of New England, Armidale, Australia

## Abstract

**Purpose:**

To advocate for a Liberal Transfusion Strategy (LTS) in neurocritical care patients with Acute Brain Injury (ABI) and provide updated evidence for optimizing transfusion thresholds in clinical guidelines.

**Background:**

Anemia frequently complicates ABI management, often necessitating red blood cell transfusions. However, the optimal hemoglobin (Hb) threshold for transfusion remains controversial. While earlier meta-analyses indicated no significant differences between LTS and restrictive transfusion strategies (RTS), emerging randomized controlled trials (RCTs) emphasize the need for reappraisal within neurocritical care.

**Methods:**

This meta-analysis included five RCTs involving 2399 patients (1,191 LTS; 1208 RTS) with ABI (subarachnoid hemorrhage, traumatic brain injury, or intracerebral hemorrhage). LTS was defined as transfusion at Hb ≤ 10–9 g/dL, and RTS as transfusion at Hb ≤ 7–8 g/dL. Outcomes assessed included sepsis or septic shock, ICU mortality, unfavorable functional outcomes at six months, venous thromboembolism (VTE), acute respiratory distress syndrome (ARDS), and in-hospital mortality.

**Results:**

RTS significantly increased the risk of sepsis or septic shock (relative risk [RR]: 1.42; 95% confidence interval [CI] 1.08–1.86; *p* = 0.01) and unfavorable functional outcomes at six months (RR 1.13; 95% CI 1.06–1.21; *p* = 0.0003). No significant differences were observed in ICU mortality (RR 1.00; 95% CI 0.84–1.20; *p* = 0.96), VTE (RR: 0.88; 95% CI 0.56–1.38; *p* = 0.58), ARDS (RR 1.05; 95% CI 0.69–1.61; *p* = 0.81), or in-hospital mortality (RR 0.98; 95% CI 0.76–1.26; *p* = 0.89). Heterogeneity was minimal (I^2^ < 25%).

**Conclusion:**

LTS demonstrates the potential to enhance safety and functional recovery in ABI patients by mitigating sepsis risk and promoting favorable neurologic outcomes. Further high-powered RCTs are warranted to validate these findings and refine transfusion protocols.

**Supplementary Information:**

The online version contains supplementary material available at 10.1186/s13054-025-05397-5.

## Introduction

Traumatic brain injury (TBI) and other forms of acute brain injury (ABI) are among the leading causes of mortality and long-term disability worldwide, affecting 25 to 70 million people yearly around the globe [1, 2]. Hemodynamic and oxygenation management are critical in brain injury care, given the brain’s vulnerability to hypoxia and ischemia [3–5]. One of the most controversial aspects of management is the choice between restrictive and liberal transfusion thresholds, which guide decisions on transfusion based on hemoglobin levels or clinical indications.

Restrictive transfusion strategies (usually < 7 g/dL) are designed to minimize the risks associated with transfusions, such as infections, immunosuppression, and volume overload [6, 7]. Conversely, liberal transfusion approaches (varies, mostly < 9–10 g/dL) aim to enhance oxygen delivery, particularly in the context of impaired cerebral autoregulation and increased oxygen demand seen in brain injury patients [8, 9]. The appropriate threshold for transfusion in this unique population remains a critical question, as evidence from non-brain injury populations may not be directly applicable [10].

Prior meta-analyses have examined transfusion thresholds in critical care and brain injury populations, with mixed findings regarding mortality and neurological outcomes [11, 12]. These analyses provided valuable insights but were limited by relatively small sample sizes and the inclusion of heterogeneous populations. Importantly, newer randomized controlled trials (RCTs) and observational studies have been published since these earlier reviews, offering additional data that may refine our understanding of the optimal transfusion strategy for brain injury patients [8, 13, 14].

In light of the evolving evidence base, we aim to conduct an updated systematic review and meta-analysis to reassess the efficacy and safety of restrictive versus liberal transfusion thresholds in brain injury patients. This review will focus on critical clinical outcomes, including mortality, neurological recovery, and secondary complications, to provide more robust and current guidance on transfusion practices in this vulnerable population.

## Materials and methods

This systematic review and meta-analysis were performed and reported under the Cochrane Collaboration Handbook for Systematic Review of Interventions and the Preferred Reporting Items for Systematic Reviews and Meta-Analysis (PRISMA) Statement guidelines [15, 16].

### Eligibility criteria

The Inclusion in this meta-analysis was restricted to studies that met all the following criteria: (1) RCTs; (2) Comparing RTS to LTS; (3) Enrolling patients who were in intensive care units; (4) With acute brain injury. In addition, studies were included if they reported any of the clinical outcomes of interest.

We excluded studies with (1) Non-related brain injury trauma patients, (2) LTS with Hb threshold at 10 g/dl or RTS with Hb threshold above 10 g/dl, and (3) LTS or RTS using a fixed hemoglobin cut-off threshold rather than a range, as we believe that using a range of hemoglobin values better reflects real-world clinical scenarios.

### Search strategies

We systematically searched PubMed, Embase, and Cochrane Central Register of Controlled Trials from inception to January 2025, with the following terms: ‘TBI’, ‘SAH’, ‘ICU’, ‘Liberal Transfusion’, ‘Restrictive Transfusion’, ‘Acute Brain Injury’, ‘Critical Care’, ‘Neurocritical’. Our search strategy was built based on the above terms as follows: (restrictive OR limited OR “restrictive transfusion” OR “Hb 7”) AND (liberal OR unlimited OR free OR “liberal transfusion” OR “Hb 10”) AND (neuro critical OR TBI OR SAH OR “acute brain” OR “brain injury”) AND (ICU OR critical care OR “intensive care”).

The preferences from all included studies, previous systematic reviews and meta-analyses were also searched manually for any additional studies. Two authors (N.N, and V.H.Q.T) independently extracted data following predefined search criteria and quality assessment. All the data in the forest plots were double-checked by the other 2 authors, N.T.B and V.N.N.D. The prospective meta-analysis protocol was registered on Prospero on January 10th, 2025; with ID (CRD42025636336).

### Outcomes of interest

Outcomes included (1) Sepsis or septic shock, (2) Unfavorable neurological outcomes at 6 months (UNOS), (3) Mortality in ICU (MICU), (4) In-hospital mortality (IM), (5) Acute respiratory distress syndrome (ARDS), and (6) Venous thromboembolism (VTE). UNOs were defined according specifically to each studies’ scale systems, further detail mentioned in Tables 1, and S3 (Supplementary). VTE events include either deep vein thrombosis or pulmonary embolism. Sepsis or Septic shock, and ARDS were defined separately in Supplementary.

**Table 1:** Characteristics of all studies met the inclusion criteria

Characteristics	HEMOTION, 2024 (N = 736)		TRAIN, 2024 (N = 820)		SAHARA, 2024 (N = 732)		Gobatto, 2019 (N = 44)		McIntyre, 2006 (N = 67)	
	LTS* (n = 369)	RTS* (n = 367)	LTS (n = 397)	RTS (n = 423)	LTS (n = 366)	RTS (n = 366)	LTS (n = 21)	RTS (n = 23)	LTS (n = 38)	RTS (n = 29)
Study design	RCT^^^		RCT		RCT		RCT		RCT	
UNOs^$^ scale, 6 months	GOS-E 1- 5		GOS-E 1–5		mRS 4–6		GOS 1–3		N/A	
LTS Hemoglobin threshold (g/dl)	≤ 10 g per deciliter		≤ 9 g per deciliter		≤ 10 g per deciliter		≤ 9 g per deciliter		≤ 9 g per deciliter	
RTS Hemoglobin threshold (g/dl)	≤ 7 g per deciliter		≤ 7 g per deciliter		≤ 8 g per deciliter		≤ 7 g per deciliter		≤ 7 g per deciliter	
ABI^+^ types	TBI		TBI, SAH, IH		SAH		TBI		TBI	
Injury-to-randomization time, days/hours (median)	55 h^&^		3 days		3 days		71 ± 38 h mean (± SD)		72 h	
Age, years (mean)	48.9	48.4	52	51	59.3	59.5	33	36	39.8	41.7
Female, %	24.1	30.5	45.1	46.6	81.7	81.7	4.7	13	26	10
Black, %	3.3	3.3	N/A	N/A	N/A	N/A	N/A	N/A	N/A	N/A
TBI, n	369	367	240	246	N/A	N/A	21	23	38	29
Heart failure, %	0.5	1.4	2.0	1.9	4.1	7.7	N/A	N/A	N/A	N/A
GCS score on admission, median	4 only motor	4 only motor	6	6	13–14	13–14	4	5	N/A	N/A

*LTS: Liberal Transfusion Strategy; RTS: Restrictive Transfusion Strategy

+ABI: Acute brain injury includes Traumatic Brain Injury (TBI), Subarachnoid Hemorrhage (SAH), and Intracerebral Hemorrhage (IH)

$UNOs at 6 months: Unfavorable Neurological Outcomes, scale systems used include Modified Rankin Scale (mRS), Glasglow Outcome Scale (GOS), or Glasglow Outcome Scale-Extended (GOS-E)

& HEMOTION RCT showed the median hours from injury to randomization similar between the two intervention groups; 55 hours for LTS, and 56 hours for RTS

n/N: number of events or patients, ^RCT: Randomized Controlled Trial

### Statistical analysis

Two authors (N.N. and V.H.Q.T.) independently extracted baseline characteristics shown in Table 1 and outcomes data based on predefined criteria for search, data extraction, and quality assessment. For all binary outcomes, we used pooled risk ratios (RR) or odds ratios (OR) with 95% confidence intervals (CIs) to compare treatment effects, and adverse events. For continuous outcomes, we applied standardized mean differences (SMDs) to account for variations in measurement scales across studies. Heterogeneity was assessed using the I^2^ statistic and the Cochrane Q test, with I^2^ > 25% and *p* < 0.10 indicating significant heterogeneity. Previously, a fixed-effect model was used when I^2^ < 25%, and a random-effects model was applied for high heterogeneity. In line with best practices outlined in the Cochrane Collaboration Handbook for Systematic Reviews of Interventions15, we employed the DerSimonian and Laird random-effects model for all analyses. This model is particularly well-suited to our meta-analysis as it accounts for both within- and between-study variability. Given that the included studies differed in their definitions of UNOs and hemoglobin transfusion thresholds, this model provides a robust framework to accommodate these variations, offering a more accurate and generalized estimate of the intervention’s effect. Statistical analyses were conducted using Review Manager (RevMan) 5.4 (Cochrane Center, The Cochrane Collaboration, Denmark).

A sensitivity analysis was also performed by N.N., removing each study from the outcome assessment. To address concerns about heterogeneity, we conducted a leave-one-out sensitivity analysis to ensure the results were not dependent on any single study. Additionally, if data permit, N.N. and D.D. will conduct two subgroup sensitivity analyses to minimize heterogeneity: (1) LTS transfusion Hb threshold (Hb ≤ 10 mg/dl vs. Hb ≤ 9 mg/dl) and (2) ABI type (TBI vs. SAH). For subgroups with missing data, a meta-regression analysis will be performed by D.D. to assess the relationship between the moderator variable and the outcome.

### Quality assessment

Two independent authors completed the risk of bias assessment (N.N, and V.H.Q.T). Disagreements were resolved through a consensus after discussing reasons for the discrepancy. If the disagreement was still not resolved, a third party (N.T.B) would be included in the discussions. For quality assessment of each randomized controlled trial (RCT), we utilized the Cochrane Collaboration’s risk of bias tool [17]. Each trial was evaluated across five domains—selection bias, performance bias, detection bias, attrition bias, and reporting bias—and assigned a risk level of high, low, or unclear. In addition, publication bias was assessed using a funnel plot of study weights and point estimates, conducted by N.N. If asymmetry was observed in the funnel plot, Egger’s test would be performed by D.D. to further evaluate the presence of publication bias.

To assess the confidence in the evidence, we exclusively included randomized controlled trials (RCTs) in our meta-analysis, excluding observational studies. As outlined in the Cochrane Handbook [15], RCTs typically provide high-certainty evidence due to their ability to minimize bias and establish causality. Nonetheless, V.H.Q.T. and N.N. independently evaluated the quality of the evidence using the Grading of Recommendation, Assessment, Development, and Evaluation (GRADE) guideline [38]. This process involved assessing potential downgrades based on five domains: risk of bias, inconsistency, indirectness, imprecision, and publication bias. Following the GRADE assessment, studies were categorized as high, moderate, low, or very low quality. A total of six distinct outcomes of interest were evaluated to determine the overall quality of the evidence across the studies.

### Updated information from previous studies

Our meta-analysis provides several novel insights that distinguish it from prior studies. First, we evaluated the risk of sepsis and septic shock, as well as acute respiratory distress syndrome (ARDS), both critical complications with significant clinical implications. Second, we incorporated the recently published randomized controlled trial (RCT), SAHARA, thereby expanding the scope of evidence. Third, we performed a secondary analysis of previously analyzed RCTs, including Robertson 2014, focusing on two pivotal outcomes: UNOs and sepsis or septic shock (Supplementary). Then, we present a detailed rationale for the exclusion of Robertson 2014 from our analysis, based on methodological discrepancies and relevance to our outcomes. These findings underscore the distinct methodological approach and enhanced scope of our meta-analysis compared to earlier investigations, providing a more comprehensive and clinically relevant synthesis of available data. Finally, we conducted a subgroup analysis including only newly published RCTs from the past year to minimize the risk of bias, as older RCTs were more likely to be influenced by potential biases.

## Results

### Study selection and baseline characteristics

**Fig. 1:**
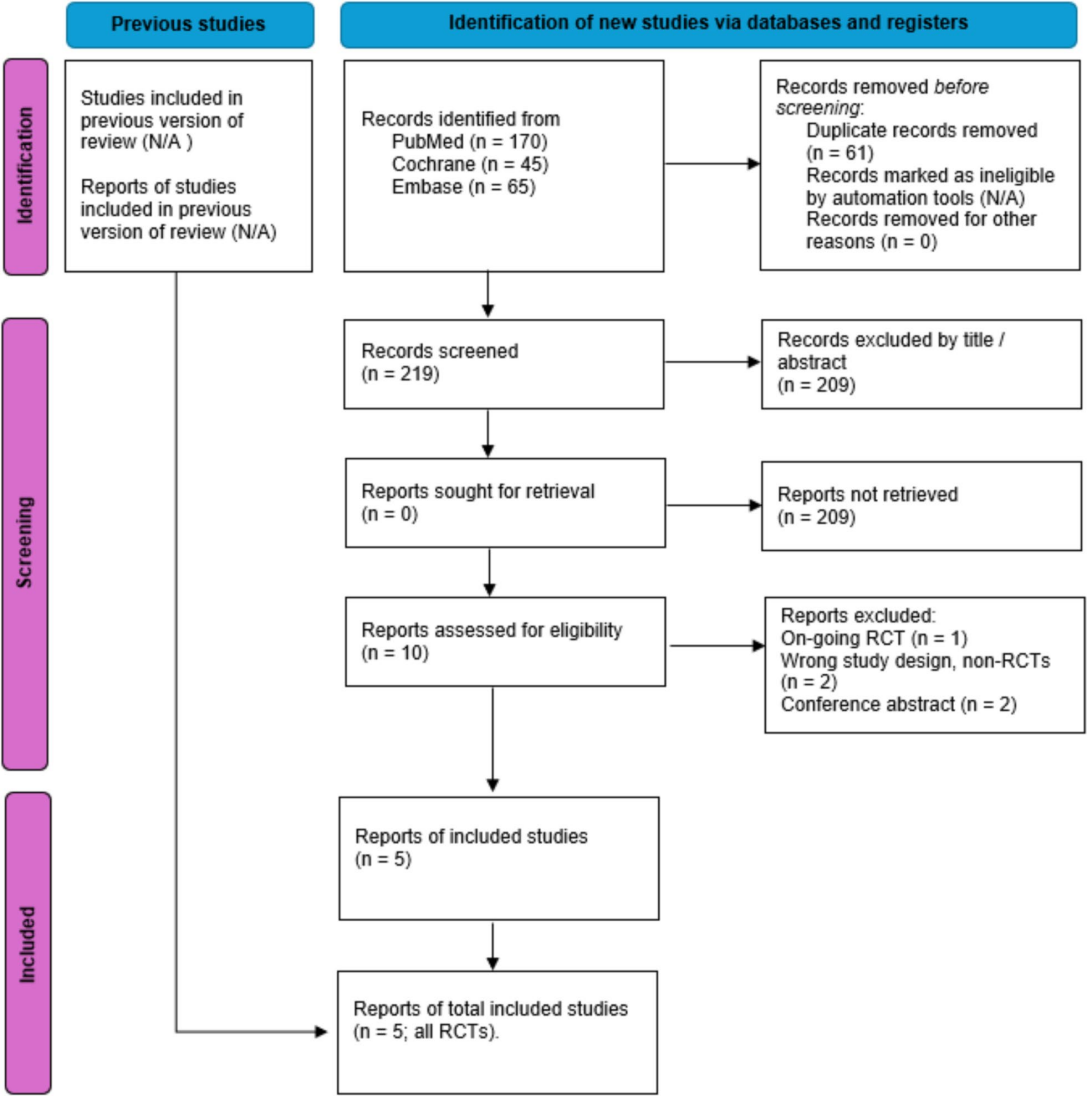
PRISMA flow diagram. PRISMA 2020 flow diagram for updated systematic reviews which included searches of databases and registers only

The initial search yielded 280 results. After the removal of duplicates by title and abstract, 10 studies remained and were fully reviewed based on the inclusion criteria. Of these, a total of 5 studies were included, comprising 2399 patients from only RCTs (Fig. 1).

A total of 1208 patients received RTS, while 1191 patients were assigned to the LTS group. The study characteristics are detailed in Table 1.

All studies evaluated TBI, and one study investigated SAH. There was significant variability across studies in the Hb threshold at which either Liberal or Restrictive transfusion was given, Hb ≤ 7–8 g per deciliter for LTS, and Hb ≤ 9–10 g per deciliter for RTS. Also, there are three different scales used to evaluate the disability outcomes at 6 months: Modified Rankin scale (mRS); Glasglow Outcomes Scale (GOS), and Glasglow Outcomes Scale-Extended (GOS-E). UNOs at 6 months were defined according to each study.

All studies reported median injury-to-randomization time after 50 h and a mean age above 30 years old. Additional details, including specific data, can be found in Table 1 below.

### Pooled analysis of all included studies

**Fig. 2:**
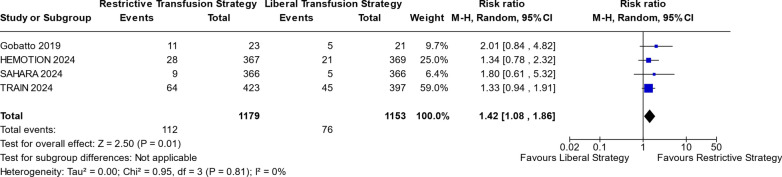
Sepsis or Septic shock risk

**Fig. 3:**
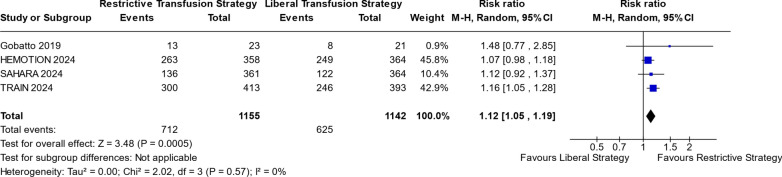
UNOs at 6-month risk

Figures 2 and 3 show the comparison between RTS and LTS groups in risk of sepsis and UNOs. For RTS, there was a noticeable increase in the risk of sepsis or septic shock, compared to LTS (RR 1.42; 95% CI 1.08–1.86; p = 0.01; I^2^ = 0%; Fig. 2). UNOs at 6 months was more likely in the RTS group compared to LTS (RR 1.12; 95% CI 1.05–1.19; p = 0.0005; I^2^ = 0%; Fig. 3).

**Fig. 4:**
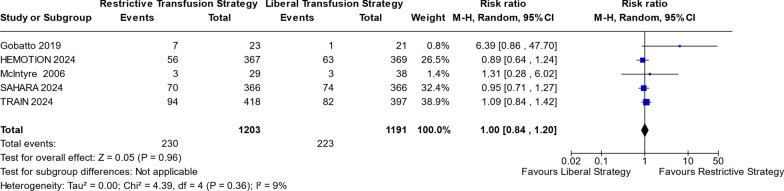
Mortality at ICU

Other outcomes, including (1) ARDS (RR 1.05; 95% CI 0.69–1.61; p = 0.81; I^2^ = 0%; Fig. S4 in Supplementary information S2), (2) VTE (RR 0.88; 95% CI 0.56–1.38; p = 0.58; I^2^ = 0%; Fig. in S5 Supplementary information S2), (3) Mortality in ICU (RR 1.00; 95% CI 0.84–1.20; *p* = 0.96; I^2^ = 9%; Fig. 4), and (4) In-hospital mortality (RR 0.98; 95% CI 0.76–1.26; p = 0.89; I^2^ = 21%; Fig. S7 in Supplementary information S2), did not differ significantly between two transfusion strategy groups. Only Figs. 2, 3, and 4 are presented below; the remaining figures are available in the Supplementary.

Figure 2 illustrates the comparison of sepsis or septic shock outcomes between RTS and LTS. The analysis reveals a statistically significant difference (*p* = 0.01), with a risk ratio greater than 1 and a 95% confidence interval (1.08; 1.86) that excludes the null value of 1. This indicates a higher risk associated with RTS compared to LTS.

Figure 3 illustrates the comparison of UNOs at 6-month outcomes between RTS and LTS. The analysis reveals a statistically noticeable difference (*p* = 0.0005), with a risk ratio greater than 1 and a 95% confidence interval (1.05; 1.19) that excludes the null value of 1. This indicates a higher risk associated with RTS compared to LTS.

Figure 4 illustrates the comparison of mortality risk in the ICU between RTS and LTS. The analysis shows no significant difference (*p* = 0.96), with a risk ratio of 1 and a 95% confidence interval (0.84; 1.20) that includes the null value of 1. These findings indicate no discernible difference in mortality risk between the two strategies.

### Rationale for exclusion of Robertson’s 2014 trial

A recently published meta-analysis11 demonstrated that RTS was comparable to LTS in UNOs at six months among ICU patients with ABI. However, our meta-analysis revealed a substantial discrepancy between these two strategies for the same outcome. To address this inconsistency, we conducted a comprehensive review of the previous meta-analysis and identified the inclusion of the Robertson et al. 2014 trial, which we had initially excluded during our study selection based on its title, and our exclusion criteria as Robertson et al.’s 2014 RCT used fixed thresholds for both LTS (10 g/dL) and RTS (7 g/dL) rather than a range of values.

Nevertheless, excluding a trial based solely on fixed cutoff points rather than a range of values may not be entirely convincing. Therefore, we present stronger justifications for our decision, supported by three key considerations: (1) Methodological differences in Robertson et al.’s study, (2) The potential biases, and (3) The impact of including or excluding Robertson et al. [23] on the meta-analysis results.

1. The methodology was carefully assessed and compared with the studies that met our inclusion criteria. Unlike recent trials, Robertson et al. [23] notably did not consider anemia as an inclusion criterion. Furthermore, both transfusion strategy groups maintained an average hemoglobin (Hb) level above 9 g/dL at all time points, limiting the study’s ability to fully capture the potential harms of a restrictive transfusion strategy (≤ 7 g/dL). This limitation is clearly illustrated in the trial’s heat maps; Supplement 2.

Additionally, the primary objectives of the study were to evaluate the effects of erythropoietin and a high transfusion threshold in acute brain injury, under the assumption that there would be minimal interaction between these two interventions. These methodological differences highlight the divergence of this trial from more recent studies in terms of methodology.

(2) A deeper investigation of the Robertson study revealed potential sources of bias, including a high rate of loss to follow-up and a relatively small sample size (approximately 100 patients per group). Notably, twelve patients in the RTS group and seven in the LTS group were excluded from the final analysis of UNOs.

We further explored the potential impact of the missing data in Robertson by performing best- and worst-case scenario analyses. In the worst-case scenario, all excluded patients were assumed to experience UNOs, yielding a borderline significant increase in UNO risk for RTS compared to LTS (RR 1.09; 95% CI 1.00–1.19; *p* = 0.05; I^2^ = 33%; Fig. S9 in Supplementary information S2). Conversely, the best-case scenario, where none of the excluded patients met the outcome, showed no significant difference between RTS and LTS (RR 1.08; 95% CI 0.97–1.20; *p* = 0.16; I^2^ = 51%; Fig. S10 in Supplementary information S2).

**Fig. 5:**
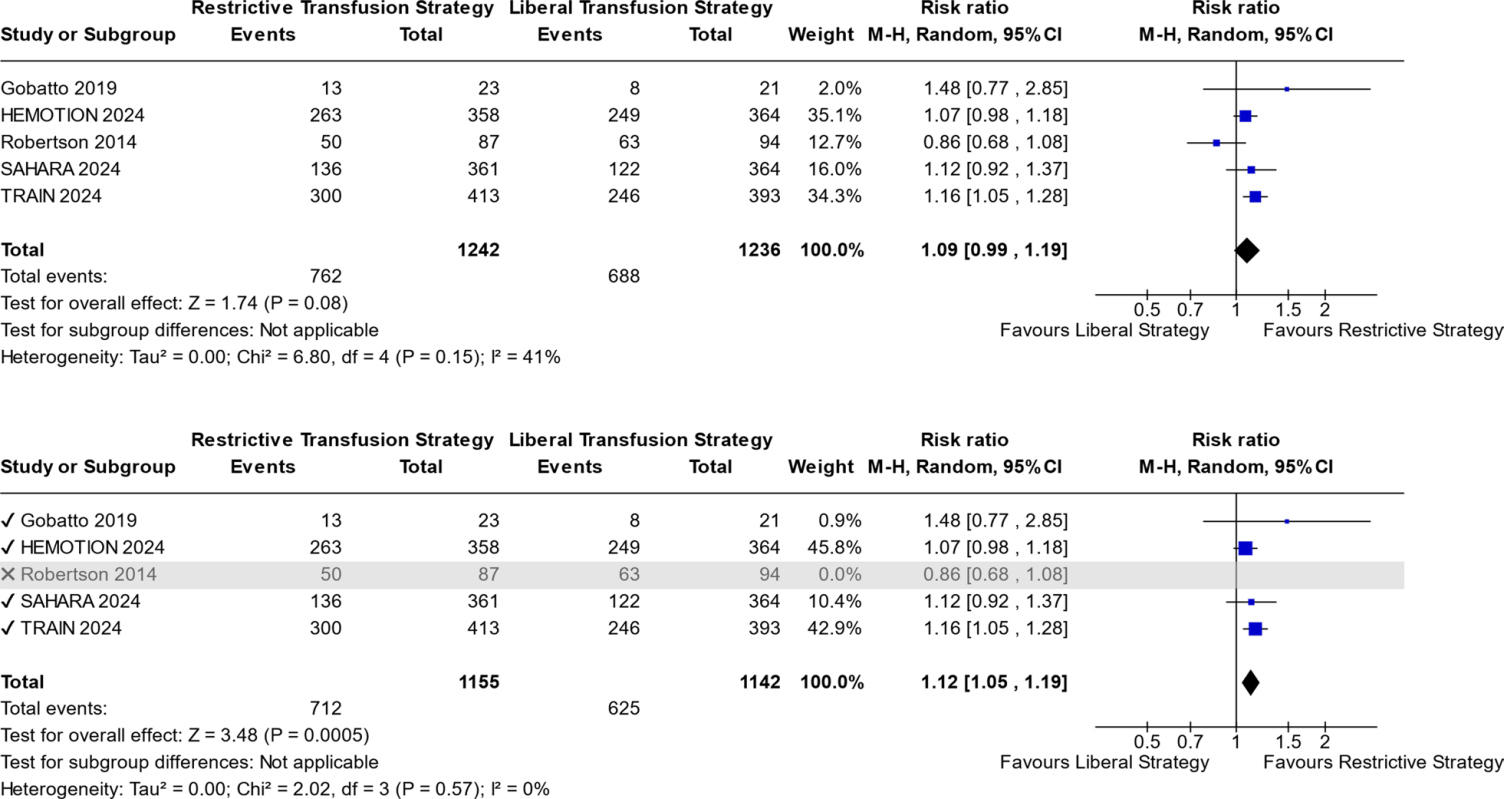
Inclusion and Exclusion of “ROBERTSON 2014” for UNOs at 6 months

(3) To reassess, we conducted a secondary analysis incorporating the Robertson trial to evaluate its impact on primary and secondary outcomes. A leave-one-out sensitivity analysis was performed using two forest plots: one including Robertson within the original dataset and another excluding it. For UNOs—a primary outcome—Robertson’s inclusion yielded findings consistent with prior meta-analyses [11, 33] (Fig. 5), whereas its exclusion revealed a distinct pattern, as detailed previously. Specifically, inclusion showed no significant difference between RTS and LTS groups for UNOs (Fig. 5). To further validate exclusion, we analyzed an alternative primary outcome—sepsis or septic shock—with complete data available. Regardless of Robertson’s inclusion, results consistently demonstrated a higher risk of sepsis or septic shock in the RTS group compared to LTS, with no detected heterogeneity (I^2^ = 0%); (RR 1.37; 95% CI 1.05–1.80; *p* = 0.02; I^2^ = 0%; Fig. S11; inclusion) and (RR 1.42; 95% CI 1.08–1.86; *p* = 0.01; I^2^ = 0%; Fig. S11; exclusion). A summary figure (Fig. S19) for highlighting the impact of inclusion and exclusion of Robertson et al. 2014 on primary outcomes could be found in Supplementary 2.

The inclusion and exclusion of the Robertson et al. trial had minimal effect on secondary outcomes. Exclusion demonstrated no substantial differences between the two transfusion strategies for ARDS (*p* = 0.76) and venous thromboembolism (*p* = 0.63) risk. Similarly, inclusion showed no statistically significant differences between LTS and RTS for either secondary outcome, with *p*-values > 0.05. In-hospital and ICU mortality were not reported in Robertson et al. [23]. Notably, moderate heterogeneity in venous thromboembolism (I^2^ > 25%) observed with inclusion was eliminated upon exclusion. All secondary outcomes for the inclusion and exclusion of the Robertson et al. trial are presented in Supplementary information S1, Fig. S20.

Figure 5 presents the impact of including and excluding the “Robertson 2014” trial on the meta-analysis of UNOs at 6 months. The upper forest plot demonstrates the inclusion of the “Robertson 2014” trial, resulting in an insignificant *p*-value (*p* = 0.08) and a 95% confidence interval (0.99; 1.19) that includes the null value (1). Conversely, the lower forest plot shows the exclusion of the trial, yielding a statistically significant result (*p* = 0.0005), with a 95% confidence interval that excludes the null value.

Based on these methodological differences and findings—including evidence of increased heterogeneity and borderline results of UNOs at 6 months in sensitivity analyses—the exclusion of the Robertson trial from our meta-analysis is a justified decision to maintain the methodological rigor and reliability of our conclusions.

### Subgroup analysis of newest RCTs (2024)

In this exploratory post hoc analysis, we evaluated only recently published randomized controlled trials (RCTs) from 2024. The rationale behind this approach was that earlier trials were small-scale, with fewer than 300 patients in each treatment arm, making them more susceptible to biases, particularly loss to follow-up. To ensure the highest quality of our findings, we focused on this subgroup for the following outcomes: (1) Sepsis or Septic Shock, (2) UNOs at 6 months, and (3) Mortality in ICU. In conclusion, our subgroup analysis included only three trials: HEMOTION, TRAIN, and SAHARA.

**Fig. 6:**
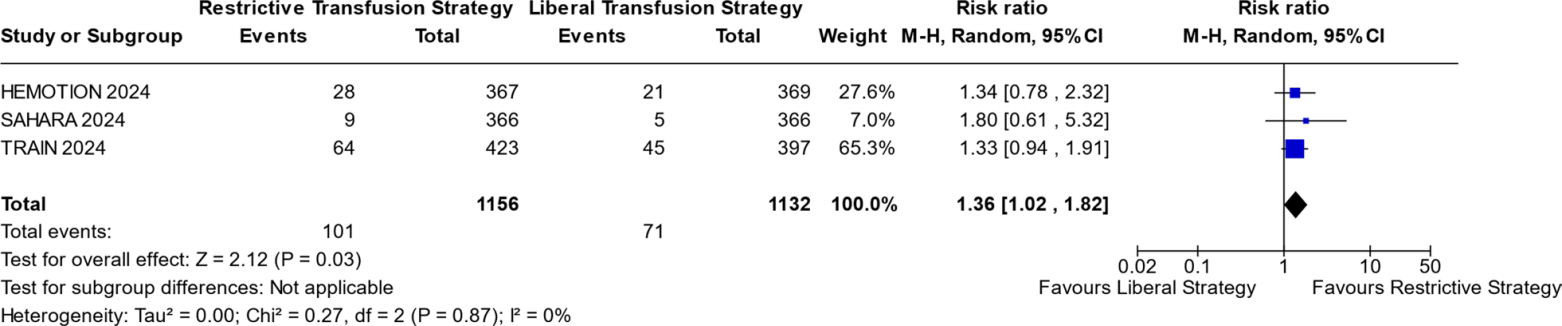
Sepsis or Septic shock risk

Our results show the inclusion of all three newest RCTs yielding consistent results as in our RESULTs section above. The RTS group had a higher risk of sepsis (RR 1.36; 95% CI 1.02–1.82; *p* = 0.03; I^2^ = 0%; Fig. 6), and UNOs at 6 months (RR 1.12; 95% CI 1.05–1.19; *p* = 0.05; I^2^ = 0%; Fig. S13) compared to LTS. For Mortality in ICU, there were no noticeable differences between the two strategies (RR 0.99; 95% CI 0.83–1.17; *p* = 0.88; I^2^ = 0%; Fig. S14). Figure 6 is shown below, while Figs. S13 and S14 can be found in Supplementary.

Figure 6 illustrates the comparison of sepsis or septic shock outcomes between RTS and LTS in RCTs 2024 subgroup. The analysis reveals a statistically significant difference (*p* = 0.03), with a risk ratio greater than 1 and a 95% confidence interval (1.02; 1.82) that excludes the null value of 1. This indicates a higher risk associated with RTS compared to LTS.

### Sensitivity analysis

Our study reports low heterogeneity, but the possibility that this could be due to limited statistical power given the small number of RCTs included (fewer than five). Nevertheless, the low heterogeneity may reflect true clinical similarity across studies, supported by the following reasons: (1) Despite the small number of studies, the total sample size is large, with over 1,000 patients per treatment strategy and a total of 2399 patients included in our meta-analysis; (2) The narrow confidence intervals observed across studies further reinforce consistency; (3) The I^2^ value remains 0 and τ^2^ = 0 consistently for our primary outcomes, indicating minimal heterogeneity; and (4) While the included studies share a similar methodology, they differ in transfusion thresholds and patient populations (TBI vs. SAH). However, we believe these variations do not limit our study but rather enhance its generalizability, as acquired brain injury (ABI) encompasses a spectrum of clinical conditions.

Despite low heterogeneity, we conducted a leave-one-out sensitivity analysis by sequentially removing one study at a time to assess whether our results were influenced by any single study. Overall, excluding individual studies did not alter the safety and efficacy endpoints, except when the TRAIN trial was removed from the pooled analysis for our primary outcomes: Sepsis or Septic Shock and Unfavorable Neurological Outcomes (UNOs) at 6 months (Figs. S17 and S18 in Supplementary information S2). The exclusion of the TRAIN trial resulted in a borderline shift for UNOs at 6 months (RR 1.09; 95% CI 1.00–1.18; *p* = 0.05; I^2^ = 0%; Fig. S18) and indicated weak differences between the two strategies for Sepsis or Septic Shock (RR 1.54; 95% CI 1.01–2.37; *p* = 0.05; I^2^ = 0%; Fig. S17). We attribute these shifts to three key factors: (1) A significant reduction in statistical power, as the removal of the TRAIN trial led to an approximate one-third decrease in the original sample size, bringing it closer to the size of our rare outcomes; (2) The broader patient population included in the TRAIN trial, which comprised not only traumatic brain injury (TBI) but also subarachnoid hemorrhage (SAH) and intracerebral hemorrhage (IH); and (3) Among the three RCTs with large sample sizes, the TRAIN trial is the only one with a stricter hemoglobin (Hb) threshold for the liberal transfusion strategy (LTS) (≤ 9 g/dL) (Table 1). The borderline shift in UNOs at 6 months may reflect a sample size effect rather than true heterogeneity, given the narrow confidence interval; however, potential heterogeneity was not entirely ruled out.

To minimize heterogeneity as much as possible, we conducted additional subgroup sensitivity analyses based on two key variations that we believe could significantly impact our primary outcomes. First, regarding the variation in *ABI types (TBI versus SAH)*, our analysis revealed that, regardless of ABI type, there was a higher incidence of UNOs at 6 months for patients receiving RTS compared to LTS. This was observed in both the TBI group (RR 1.10; 95% CI 1.02–1.19; *p* = 0.01; I^2^ = 0%; Fig. S21 in Supplementary information S1) and the SAH group (RR 1.15; 95% CI 1.03–1.28; *p* = 0.01; I^2^ = 0%; Fig. S21 in Supplementary information S1).

Second, regarding the variation in *LTS threshold (Hb* ≤ *10 mg/dl versus Hb* ≤ *9 mg/dl)*, our subgroup analysis showed a higher risk of UNOs at 6 months with RTS compared to LTS with Hb ≤ 9 mg/dl (RR 1.17; 95% CI 1.06–1.28; *p* = 0.002; I^2^ = 0%; Fig. S22 in Supplementary information S1), and a borderline result for the LTS group with Hb ≤ 10 mg/dl (RR 1.08; 95% CI 1.00–1.97; *p* = 0.06; I^2^ = 0%; Fig. S22 in Supplementary information S1).

A similar pattern was observed for sepsis or septic shock, where RTS was associated with a higher incidence compared to LTS set at Hb ≤ 9 mg/dl (RR 1.41; 95% CI 1.02–1.97; *p* = 0.04; I^2^ = 0%; Fig. S23 in Supplementary information S1). In contrast, no statistically significant difference in the composite risk of sepsis was found between the two transfusion strategies for the LTS group with a threshold of Hb ≤ 10 mg/dl (RR 1.42; 95% CI 0.87–2.32; *p* = 0.16; I^2^ = 0%; Fig. S23 in Supplementary information S1).

We were initially surprised by these findings and conducted a thorough investigation into the discrepancy between LTS thresholds of Hb ≤ 10 g/dL and Hb ≤ 9 g/d. Notably, we discovered that the ratio of TBI to SAH patients was 1.0 in the Hb ≤ 10 g/dl LTS group, whereas the ratio was 1.5 for the Hb ≤ 9 g/dl LTS group, indicating a predominance of TBI patients. No data were available for a separate subgroup sensitivity analysis of sepsis or septic shock based on ABI type. Nevertheless, meta-regression analysis assessing the association between sepsis composite outcomes and ABI type (Fig. S24 in Supplementary information S1) revealed no statistically significant relationship (*p* = 0.905). This finding is likely limited by the low variability in ABI type, as most included studies focused exclusively on TBI.

In conclusion, we hypothesize that the higher risk of sepsis or septic shock, and UNOs in the RTS group may be more pronounced in TBI patients compared to LTS with Hb ≤ 9 g/dL, whereas this pattern may differ in the SAH group. Future research should explore the impact of higher LTS thresholds (Hb ≤ 10 g/dL) and the specific role of SAH subgroups in primary outcome risks.

### Quality assessment

The Risk of Bias (RoB 2) [17] tool was used for quality assessment. No studies are at high risk for biases (Table S2 in Supplementary information S2). According to the Cochrane Handbook, RCTs generally provide high-certainty evidence due to their ability to minimize bias and establish causality. Nevertheless, the GRADE assessment designated mortality at ICU as low-quality evidence due to wide confidence intervals and the risk of publication bias. Three outcomes—in-hospital mortality, venous thromboembolism risk, and acute respiratory distress syndrome—were classified as moderate-quality evidence. In contrast, only two out of six assessed outcomes, sepsis or septic shock and unfavorable neurological outcomes at six months, were rated as high-quality evidence (GRADE Assessment, Supplementary information S1).

A funnel plot (Fig. S15 in Supplementary information S2) was used to assess publication bias. While large-scale trials were symmetrically distributed at the top, small-sized studies exhibited an asymmetric distribution, suggesting potential publication bias. This may be attributed to the inherent limitation of smaller sample sizes, which are less likely to yield positive outcomes due to reduced statistical power to detect differences between intervention groups. Consequently, such studies may be less likely to be published. Nonetheless, to further evaluate potential bias, we conducted Egger’s test. Contrary to expectations, the p-values for all outcomes were above 0.05, indicating no significant risk of publication bias (Table S6 in Supplementary information S1).

## Discussion

This updated systematic review and meta-analysis of five randomized controlled trials involving 2,394 patients with ABI compared restrictive transfusion strategies with liberal transfusion strategies. Our findings indicate that RTS is associated with a higher risk of sepsis or septic shock and worse neurological outcomes at six months compared to LTS. Importantly, no significant differences were observed between the two strategies in terms of ICU mortality, in-hospital mortality, venous thromboembolism, or acute respiratory distress syndrome. These results suggest that LTS may offer advantages in reducing infections and improving neurological recovery while maintaining comparable safety in terms of mortality and systemic complications in neurocritical care patients.

The current evidence on transfusion thresholds in neurocritical care remains inconclusive, with earlier meta-analyses reporting no significant differences between restrictive transfusion strategies and liberal transfusion strategies [11, 12], particularly in mortality outcomes. While these meta-analyses suggested uniformity in pooled results, findings from individual observational studies and randomized trials have been inconsistent, with some studies favoring LTS and others favoring RTS for specific outcomes. These discrepancies may be attributed to methodological variability and lack of randomization, heterogeneity in patient populations, and small sample sizes. By incorporating newer trials and a larger pooled dataset, our study identified statistically significant differences in outcomes such as sepsis risk and neurological recovery, suggesting potential benefits of LTS in these domains. The clinical implication of these statistical significance remains uncertain.

In neurocritical patients, where compensatory mechanisms for maintaining oxygenation such as cerebrovascular reactivity are impaired, liberal transfusion may address this physiologic need by enhancing oxygen delivery in critical settings. This is supported by findings from the TRAIN trial, which observed a decreased risk of cerebral ischemic events in the liberal transfusion group, potentially explaining the observed neurological protection associated with LTS. While findings from the HEMOTION trial, which showed no significant differences in mortality but demonstrated better scores in functional independence and quality of life among survivors in the liberal transfusion group, suggest benefits of LTS, these results predominantly reflect TBI populations.

Four of the five trials included in our analysis focused primarily on patients with TBI, while only one trial exclusively enrolled patients with SAH. This disparity reflects the predominance of TBI in the current literature on transfusion thresholds and may limit applicability for specific neurocritical subpopulations. As such, findings should be interpreted with caution, as liberal transfusion carries risks, including an increased likelihood of progressive cerebral hemorrhagic injury [18], transfusion reactions, and cerebral vasospasm [19], which may be more pronounced in certain subpopulations such as SAH.

Our pooled results suggest that LTS may mitigate sepsis risk, although the underlying mechanisms remain unclear. A possible explanation is that anemia has been linked to immune dysfunction due to inflammation-induced erythropoietin suppression [20], oxidative stress [21], and nutrient deficiencies; prior studies have also suggested that transfusions themselves may increase infection risk [10, 22]. These highlight the systemic implications of anemia in a critically ill patient.

Furthermore, our findings align with the potential role of anemia in increasing sepsis risk. Evidence suggests that anemia is associated with a higher likelihood of invasive bacterial infections, possibly due to impairments in both innate and adaptive immune responses, as well as increased gut permeability [26, 27]. Historically, anemia has been recognized as a risk factor for infections, including tuberculosis [28] (TB). Additionally, a recent systematic review found that lower baseline hemoglobin levels and a progressive decline in hemoglobin during early sepsis were linked to higher mortality risk [29]. In patients with traumatic brain injury (TBI), multiple factors contribute to sepsis risk. Notably, both anemia and TBI share a common underlying mechanism—transient immunosuppression. Following TBI, a systemic anti-inflammatory response leads to suppression of cell-mediated immunity, while increased intestinal permeability facilitates bacterial translocation, particularly of pathogens such as Escherichia coli [30]. Given the frequent coexistence of anemia and TBI [31], the immunosuppressive burden is compounded, potentially diminishing the host’s ability to mount an effective immune response against infections. Based on these pathophysiological insights, we hypothesize that greater severity of anemia is associated with a higher susceptibility to infections, and this vulnerability is further exacerbated in the setting of TBI. Collectively, these findings may indirectly support the observed association between a restrictive transfusion strategy and a heightened risk of sepsis compared to a liberal transfusion strategy.

Based on the relationship between anemia and sepsis, we hypothesize that LTS may be preferable to RTS in ABI management. Given the complex interplay between these conditions, we propose two overlapping phases in ABI patients. The initial phase is characterized by an inflammatory response triggered by tissue damage and hypoxia [35]due to reduced oxygen perfusion. If left uncorrected, this may further escalate systemic inflammation. Simultaneously, post-ABI immune suppression—part of the body’s natural response to exaggerated inflammation—dampens immune regulation [36], prolonging the inflammatory process.

In the later stages, transient immunosuppression may promote gut bacterial translocation [30], increasing susceptibility to sepsis. Initiating transfusion earlier at a higher hemoglobin (Hb) threshold could help disrupt this cascade, potentially reducing sepsis risk by preventing the progression of these pathophysiological events. Additionally, recent retrospective data [37] suggest that earlier transfusion at a higher Hb threshold may improve short-term survival in sepsis and septic shock. Nevertheless, further research is needed to better understand the interplay between sepsis, anemia-induced brain injury (ABI), and low Hb thresholds.

At the beginning of this year, a meta-analysis [33] in *Critical Care Medicine* reported a higher incidence of ARDS with LTS, whereas our study found no significant difference in ARDS risk between LTS and RTS. This discrepancy may stem from: (1) our broader patient population, including ABI cases (TBI, SAH, and ICH), compared to the prior study’s TBI-only focus; (2) the inclusion of the Robertson trial in their analysis, which was excluded from ours; and (3) our higher LTS threshold (Hb ≤ 10 g/dL vs. < 9 g/dL). Moreover, the meta-analysis found no statistical difference in neurological outcomes between transfusion strategies. However, after excluding the Robertson trial in their sensitivity analysis, the difference became noticeable, reinforcing our findings in UNOs. Regarding ARDS, we hypothesize that a lower transfusion threshold leads to a greater number of transfused blood units. This increased transfusion volume, along with the associated inflammatory response, may elevate the risk of ARDS [34]. Nonetheless, ICU clinicians should balance the risks and benefits of LTS to optimize patient outcomes while minimizing transfusion-related complications.

Further, our findings also support the growing consensus in the literature that transfusion strategies should be individualized. Neurocritical patients are a heterogeneous group, with variations in injury type, comorbidities, and vascular reserve. A one-size-fits-all approach to transfusion is unlikely to be effective, and thresholds should instead be tailored based on patients’ unique clinical picture. As a final point in Discussion, although low heterogeneity was observed in our study, the statistical power to detect meaningful differences is limited by the small number of included trials (only five). As a result, the possibility of undetected heterogeneity due to insufficient power cannot be entirely ruled out.

### Limitations

Our study has certain limitations. First and foremost, our findings may be influenced by potential confounders, including baseline hemoglobin levels, underlying comorbidities, and injury severity. To mitigate these confounding effects, we deliberately selected randomized controlled trials (RCTs), as they are inherently less susceptible to confounding compared to observational studies. In addition, we conducted a rigorous risk-of-bias assessment for each included trial and found a low risk of bias related to randomization. However, it is important to acknowledge that each RCT implements randomization strategies to control for specific confounders. In the Gobatto 2019 trial, while randomization was performed, it did not explicitly account for the key confounders mentioned above, which may have influenced the outcomes. Consequently, in our post hoc analysis, we prioritized more recent trials (HEMOTION, TRAIN, and SAHARA), as these studies explicitly considered hemoglobin levels at randomization, pre-existing comorbidities, and injury severity during the randomization process (Table S4 in Supplementary information S1). The findings from the subgroup analysis of the 2024 RCTs remain consistent with our overall results, further reinforcing the robustness of our conclusions and supporting the notion that our findings are minimally affected by confounding effects. Moreover, our post-hoc analysis may introduce a potential risk of false-positive findings, which is partially mitigated by the use of a random-effects model. Nevertheless, as no adjustments were made for multiple comparisons, these post-hoc analyses should be considered exploratory.

While randomized trials reduce confounding, variations in ICU management practices and randomization strategies across studies may still impact the findings. ICU and in-hospital mortality rates could be influenced by differences in patient management protocols among ICU units in different trials. Notably, the original studies did not provide detailed information on whether all ICUs adhered to a standardized management protocol beyond the restrictive transfusion strategy (RTS) and liberal transfusion strategy (LTS). The lack of uniformity in patient management may have introduced additional confounding factors, which were not specifically addressed in our analysis.

The sample size remains relatively small, particularly for rare outcomes such as VTE, ARDS, and In-hospital Mortality, limiting the ability to detect subtle differences between strategies. Variability in the definitions of RTS and LTS across trials may have introduced heterogeneity into the analysis. Additionally, our focus on randomized trials excluded observational studies that could provide valuable real-world insights. Most of the included studies assessed short- to medium-term outcomes, leaving uncertainty about long-term effects on recovery and prognosis. Finally, the findings may not be generalizable to all neurocritical populations, as the majority of included patients had TBI, with limited representation of other types of acute brain injury.

## Conclusion

This meta-analysis of randomized controlled trials, including 2,394 patients with ABI, demonstrates the potential benefits of liberal transfusion strategies, showing non-inferior mortality outcomes compared to a restrictive approach. Liberal transfusion was associated with a reduced risk of sepsis or septic shock and improved neurological recovery at six months, with no significant differences in ICU mortality, in-hospital mortality, venous thromboembolism, or acute respiratory distress syndrome. Notably, it decreased the incidence of unfavorable neurological outcomes across all types of ABI and resulted in fewer composite sepsis outcomes, particularly in patients with traumatic brain injury (TBI). Overall, this study supports the consideration of a liberal transfusion strategy as a viable approach in neurocritical care and emphasizes the need for individualized transfusion protocols tailored to the specific needs of patients.

## Statement

The PRISMA 2020 statement [32] includes a 27-item checklist covering the introduction, methods, results, and discussion sections of a systematic review report. Our systematic review and meta-analysis adhere to the core requirements outlined in the PRISMA checklist (Table S4 in Supplementary information S1).

## Supplementary Information

Additional file 1.

Additional file 2.

## Data Availability

All data generated or analysed during this study are found in following published Randomized Controlled Trials: HEMOTION [13], TRAIN [8], SAHARA [14], Gobatto et al. [24], Robertson et al. [23], McIntyre et al. [25] (REFERENCES)
