# Supplementary material for: Liberal transfusion strategies reduce sepsis risk and improve neurological recovery in acute brain injury: an updated systematic review and meta-analysis
Source: Crit Care. 2025 May 6;29:181. doi: 10.1186/s13054-025-05397-5 (PMC12057087; doi:10.1186/s13054-025-05397-5)
Supplement: Supplementary file 1 — Additional file 1. [file 13054_2025_5397_MOESM1_ESM.docx]

**GRADE ASSESSMENT**

| **RTS compared to LTS for ABI** | | | | | |
| --- | --- | --- | --- | --- | --- |
| **Patient or population:** ABI  **Setting:** Intensive Care Units  **Intervention:** RTS  **Comparison:** LTS | | | | | |
| **Outcomes** | **№ of participants (studies) Follow-up** | **Certainty of the evidence (GRADE)** | **Relative effect (95% CI)** | **Anticipated absolute effects** | |
|  |  |  |  | **Risk with LTS** | **Risk difference with RTS** |
| Sepsis or Septic shock risk. | 2332 (4 RCTs) | ⨁⨁⨁⨁ High | **RR 1.42** (1.08 to 1.86) | 66 per 1,000 | **28 more per 1,000** (5 more to 57 more) |
| Unfavorable Neurological Outcomes (UNOs) | 2297 (4 RCTs) | ⨁⨁⨁⨁ High | **RR 1.12** (1.05 to 1.19) | 547 per 1,000 | **66 more per 1,000** (27 more to 104 more) |
| Mortality at ICU | 2394 (5 RCTs) | ⨁⨁◯◯ Low^a,b^ | **RR 1.00** (0.84 to 1.20) | 187 per 1,000 | **0 fewer per 1,000** (30 fewer to 37 more) |
| Acute Respiratory Distress Syndrome (ARDS) risk | 1596 (3 RCTs) | ⨁⨁⨁◯ Moderate^a^ | **RR 1.05** (0.69 to 1.61) | 50 per 1,000 | **2 more per 1,000** (15 fewer to 30 more) |
| Venous thromboembolism risk | 1596 (3 RCTs) | ⨁⨁⨁◯ Moderate^a^ | **RR 0.88** (0.56 to 1.38) | 50 per 1,000 | **6 fewer per 1,000** (22 fewer to 19 more) |
| In-hospital mortality | 1579 (5 RCTs) | ⨁⨁⨁◯ Moderate^a^ | **RR 0.98** (0.76 to 1.26) | 208 per 1,000 | **4 fewer per 1,000** (50 fewer to 54 more) |
| ***The risk in the intervention group** (and its 95% confidence interval) is based on the assumed risk in the comparison group and the **relative effect** of the intervention (and its 95% CI).  **CI:** confidence interval; **RR:** risk ratio | | | | | |
| **GRADE Working Group grades of evidence** **High certainty:** we are very confident that the true effect lies close to that of the estimate of the effect. **Moderate certainty:** we are moderately confident in the effect estimate: the true effect is likely to be close to the estimate of the effect, but there is a possibility that it is substantially different. **Low certainty:** our confidence in the effect estimate is limited: the true effect may be substantially different from the estimate of the effect. **Very low certainty:** we have very little confidence in the effect estimate: the true effect is likely to be substantially different from the estimate of effect. | | | | | |

#### Explanations

a. 95% CI does not exclude RR of 1

b. Funnel plot (figure 15) shows potential publication bias in smaller studdies

**Table 6. Egger’s Test and Begg’s Test**

| Outcome | Egger’s Test P-value |
| --- | --- |
| Sepsis or septic shock risk | 0.1164 |
| UNOs | 0.5695 |
| ARDS | 0.5927 |
| VTE | 0.2570 |
| ICU Mortality | 0.1827 |
| In-hospital Mortality | 0.0973 |

Table 6. Egger’s test for publication bias found no statistically significant publication bias for UNOs (P=0.5695), ARDS (P=0.5927), VTE (P=0.2570) or mortality in the ICU (P=0.1827), sepsis or septic shock risk (P=0.1164) and in-hospital mortality (P=0.0973).

**Figure 19**. Impact of Robertson Trial Inclusion/ Exclusion on **Primary Outcomes** Analysis

**UNOs at 6 months**

**A.**


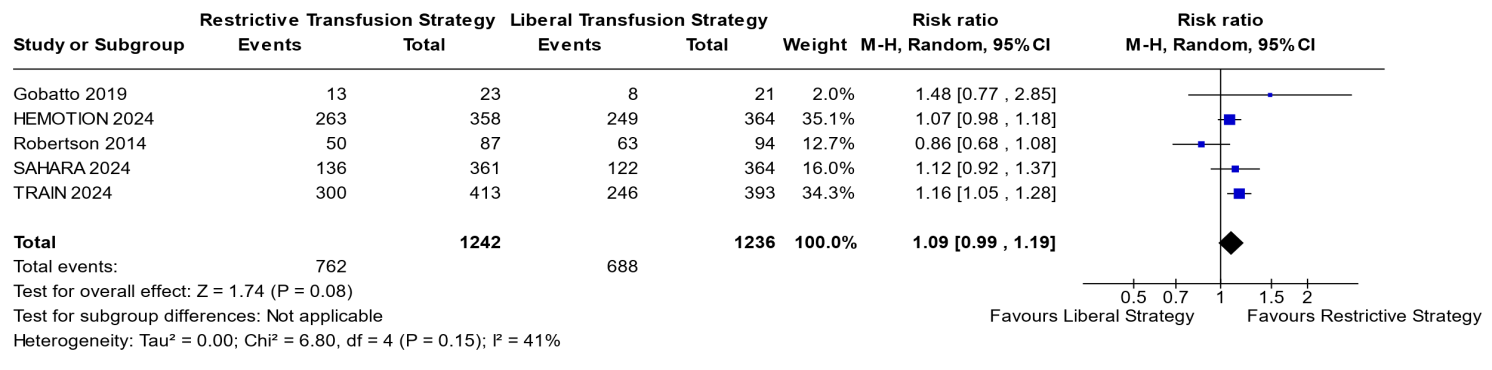
 **B.**


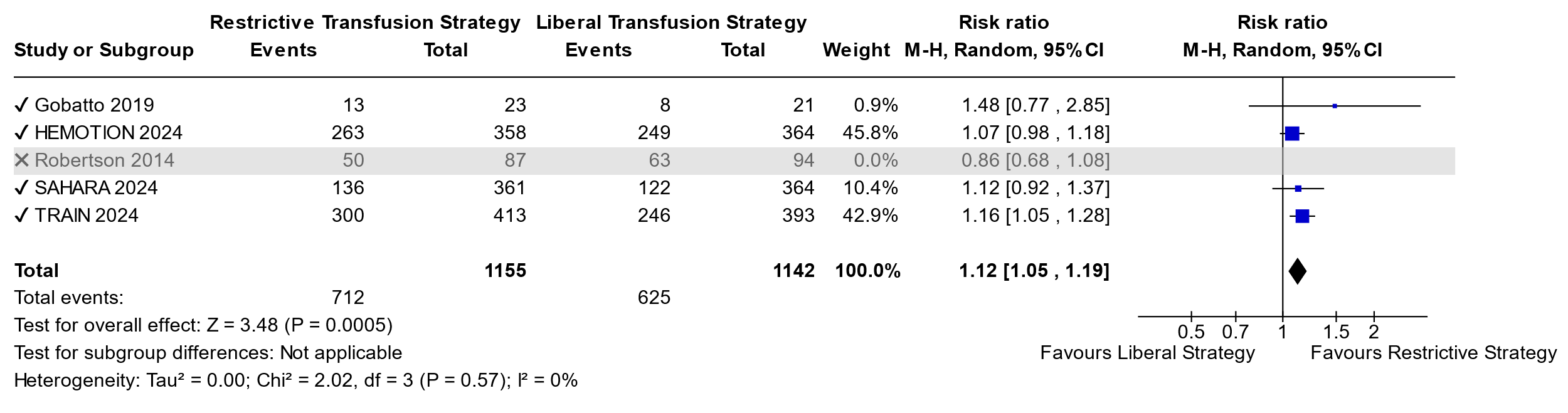


**Sepsis or Septic Shock**

**A.**


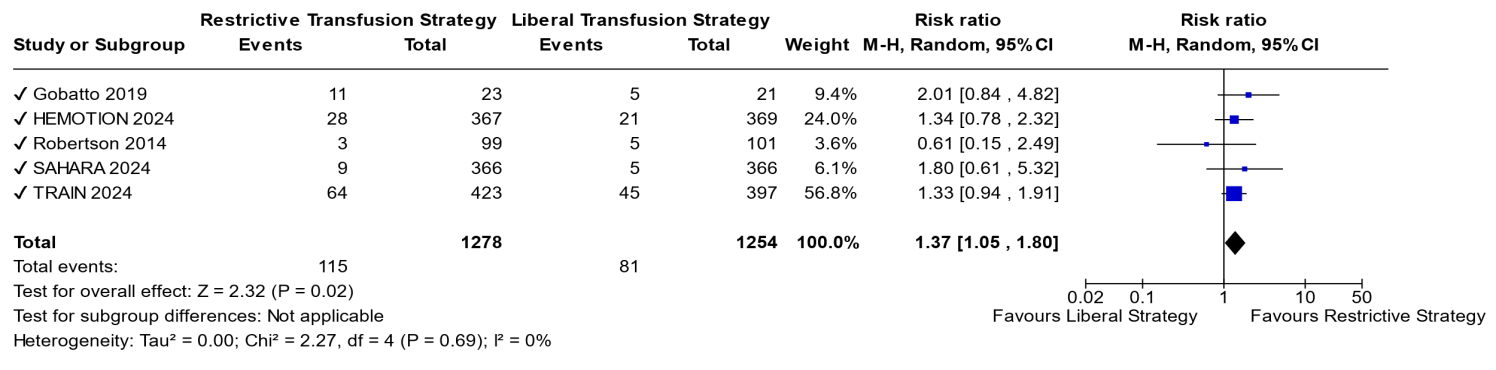


**B.**


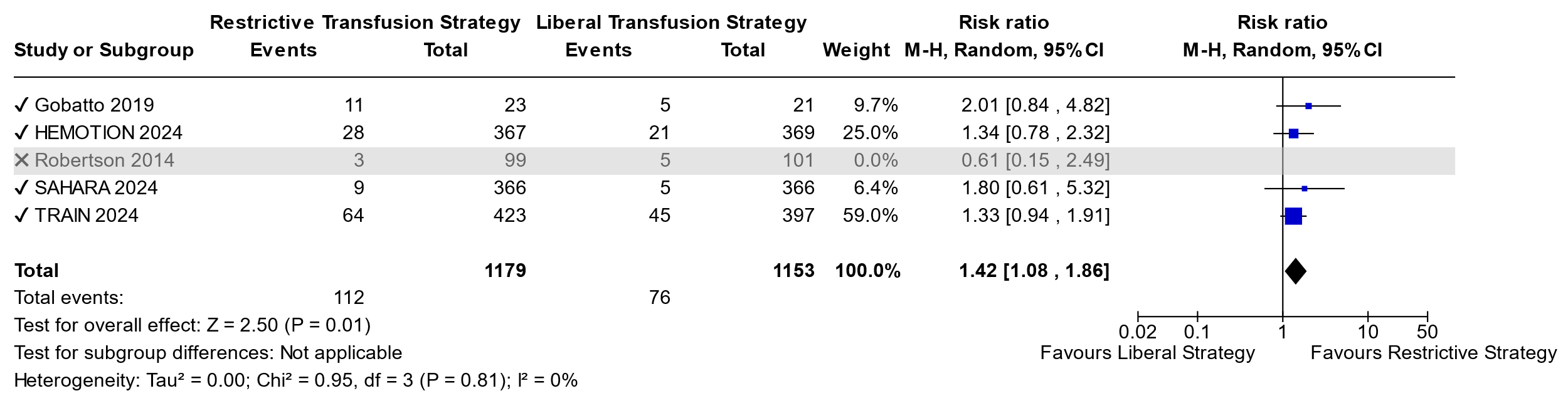
**Figure 19.** The inclusion (A) and exclusion (B) of the Robertson et al. trial impacted meta-analysis outcomes. For **UNOs at 6 months**, exclusion shifted **p = 0.08** (A) to **p = 0.0005** (B), strengthening significance. Similarly, **Sepsis or Septic Shock** became statistically significant upon exclusion (**p = 0.01**). This underscores the trial's influence on overall conclusions.

Figure 20. Impact of Robertson Trial Inclusion/ Exclusion on **Secondary Outcomes Analysis**.

**Acute Respiratory Distress Syndrome (ARDS)**

**A.**


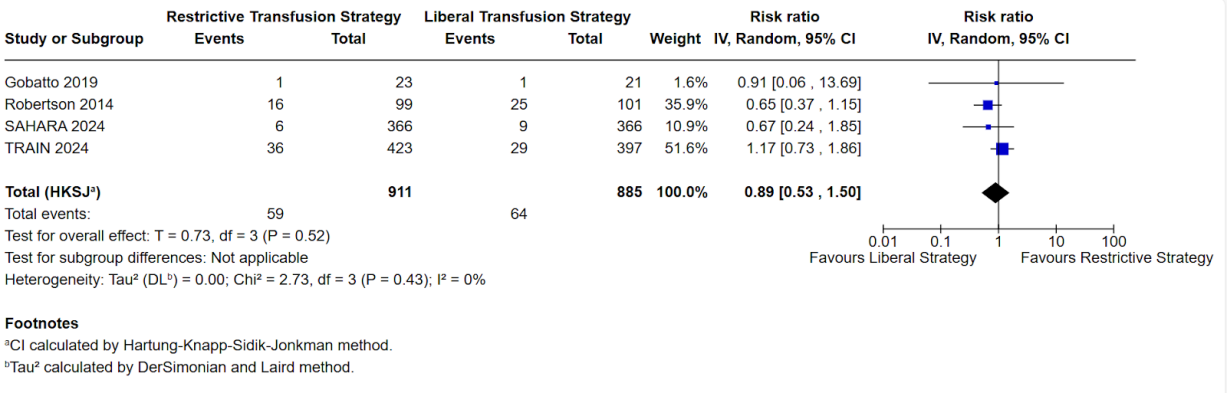


**B.**


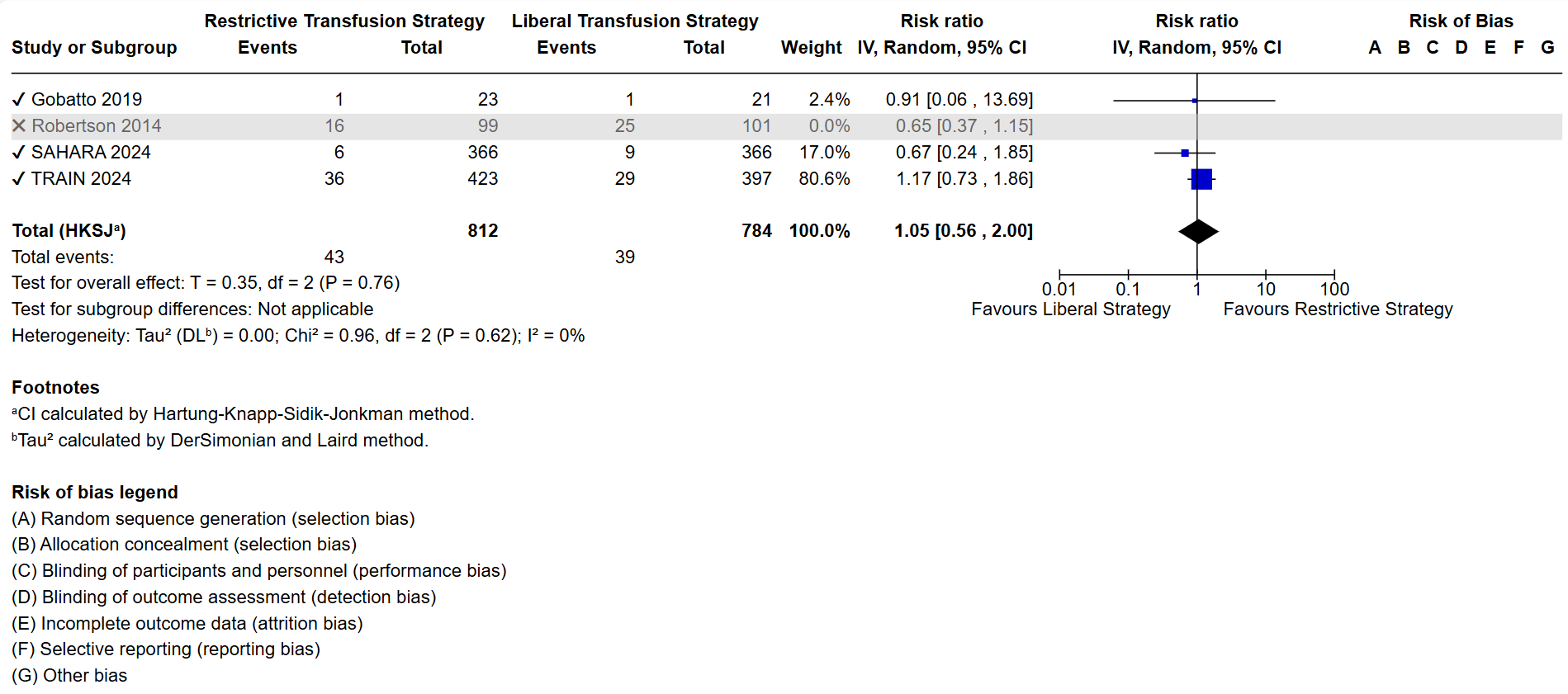


**Venous Thromboembolism**

**A.**

**
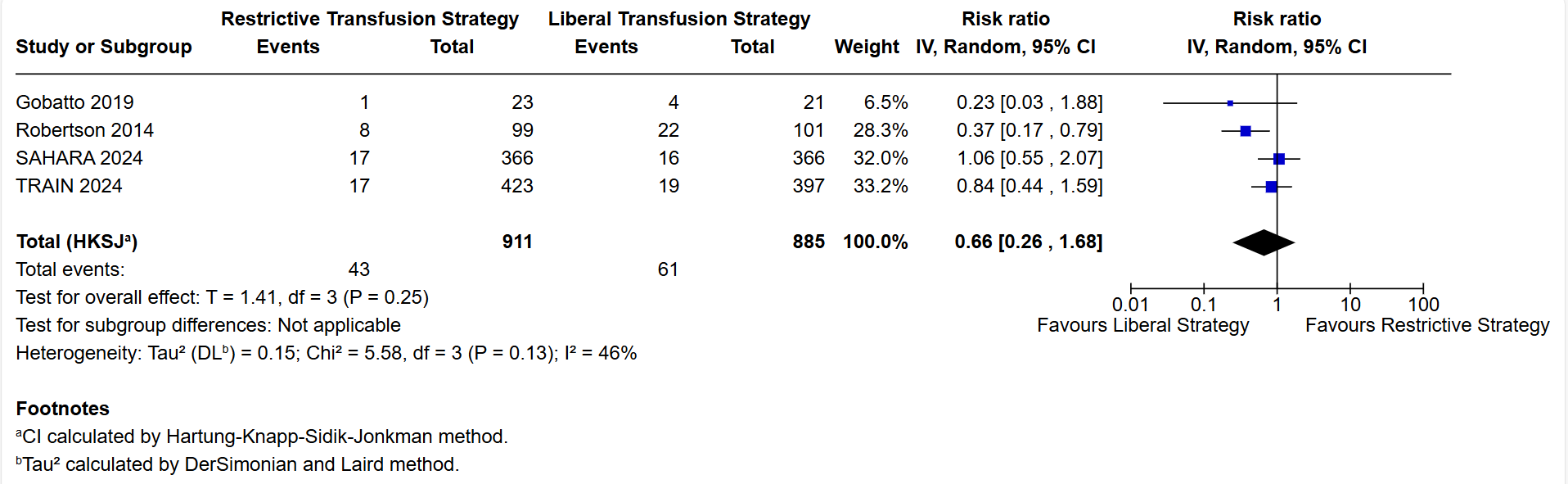
**

**B.**

**
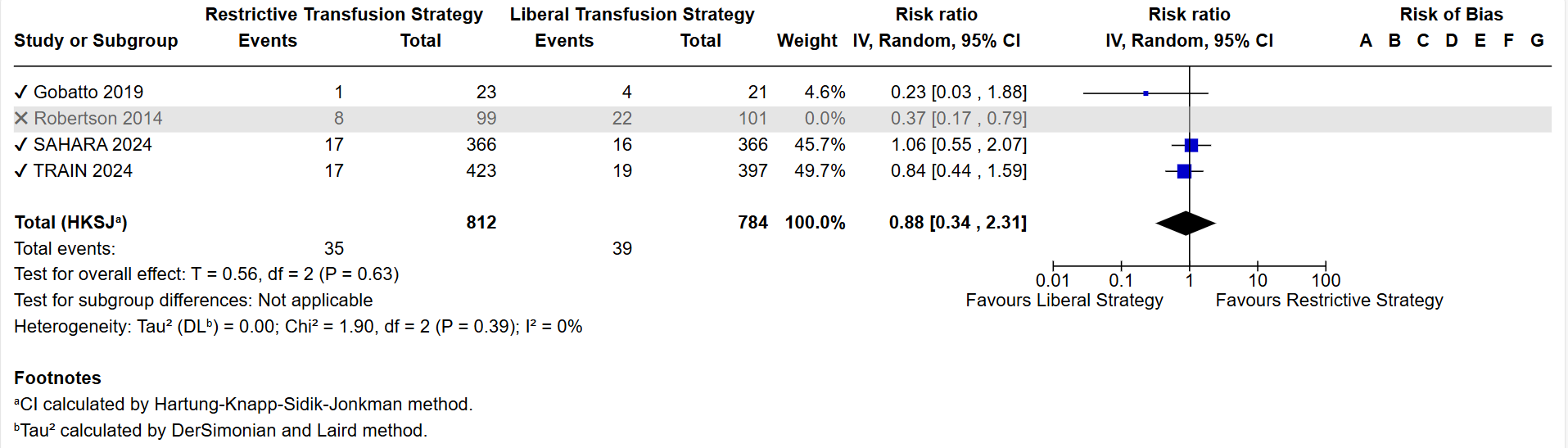
**

**Figure 20.** The inclusion (A) and exclusion (B) of the Robertson et al. trial had minimal impact on secondary outcomes. **ARDS (p = 0.76) and Venous Thromboembolism (p = 0.63)** showed no significant shifts. Similarly, inclusion (A) showed no statistical difference between LTS and RTS, with **p-values > 0.05**. **In-hospital and ICU mortality** were not reported in Robertson et al. (2014). Notably, moderate heterogeneity in **Venous Thromboembolism (I² > 25%)** with inclusion was eliminated after exclusion.

**Figure 21. Impact of Transfusion Strategy on UNOs Incidence in Acute Brain Injury: A Subtype-Specific Analysis**

**UNOs at 6 months**

**A.**
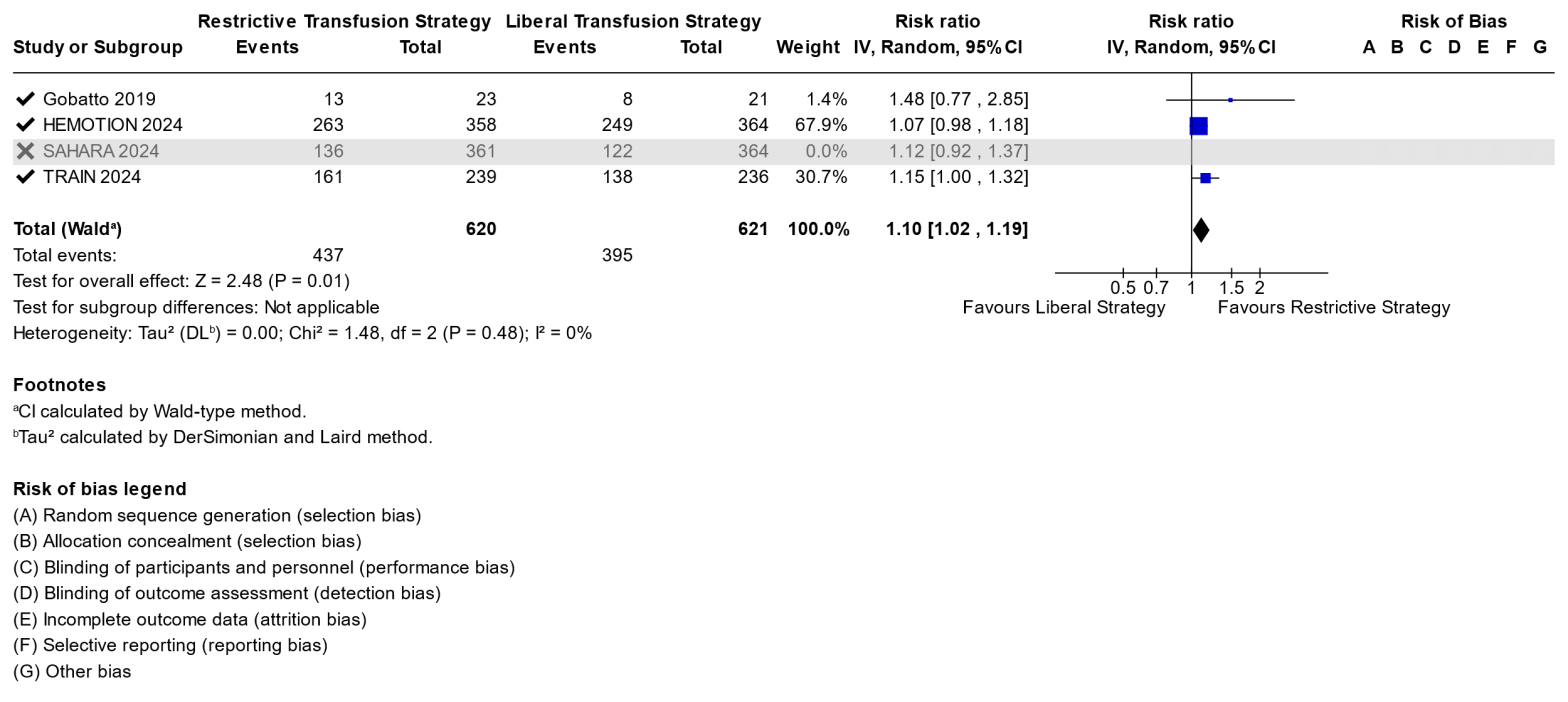
**B.**


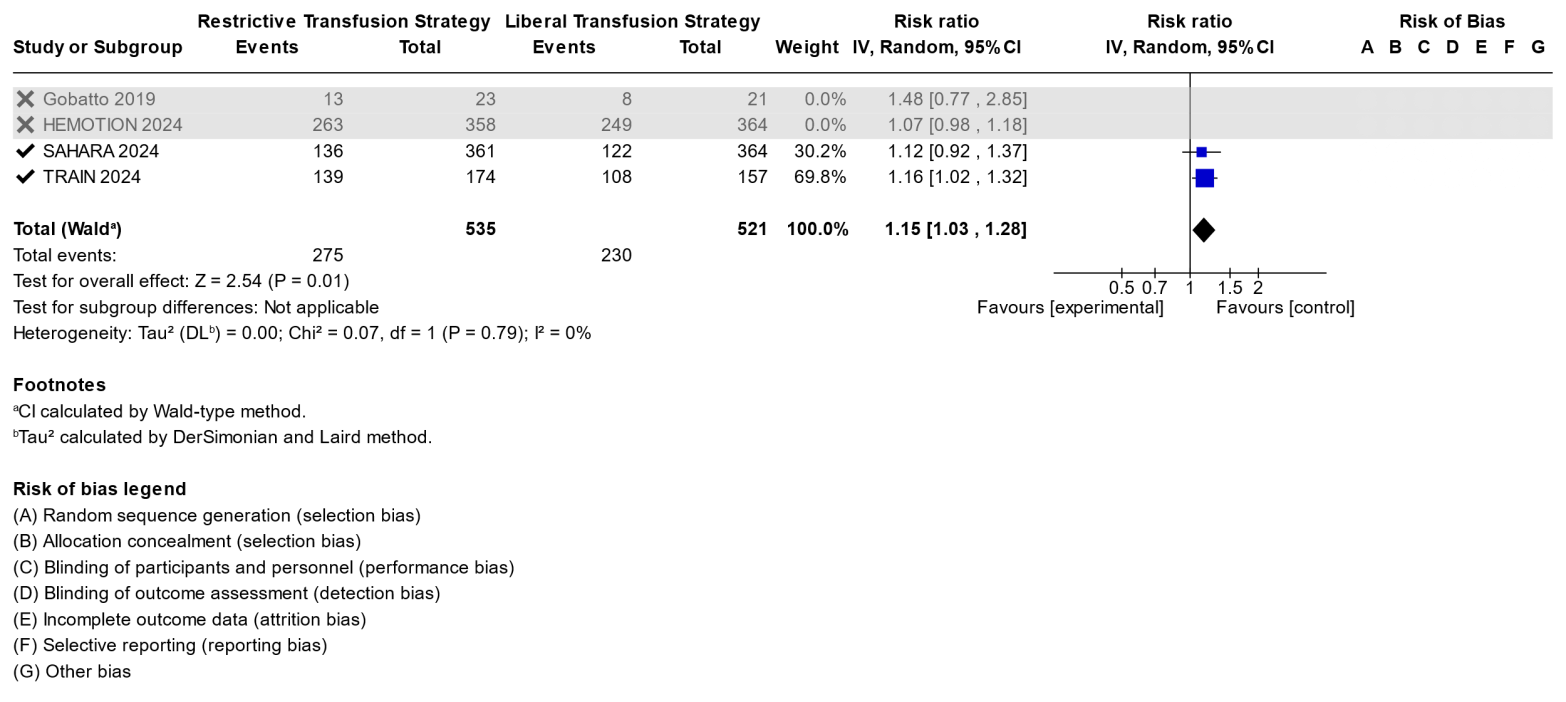


Figure 21 illustrates a higher incidence of UNOs at 6 months in patients who received the Restrictive Strategy compared to those under the Liberal Strategy, regardless of Acute Brain Injury type. Both the (A) Traumatic Brain Injury and (B) Subarachnoid Hemorrhage groups show statistically significant differences between the two transfusion strategies, with a p-value of 0.01 for both.

**Figure 22. Impact of Transfusion Strategy on UNOs Incidence, Stratified by Liberal Transfusion Threshold (Hb ≤ 9 g/dL vs. Hb ≤ 10 g/dL)**

**UNOs at 6 months**

**A.
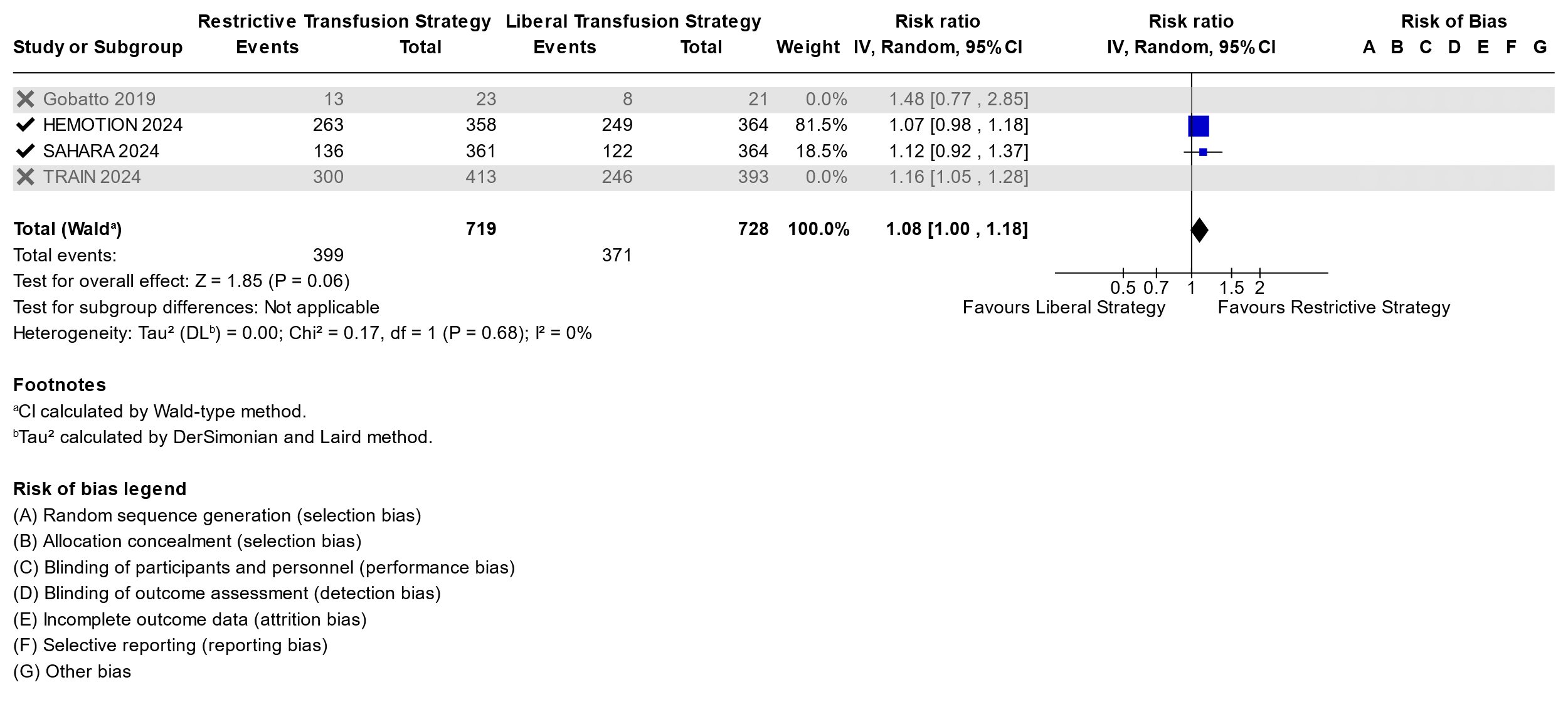
B.**
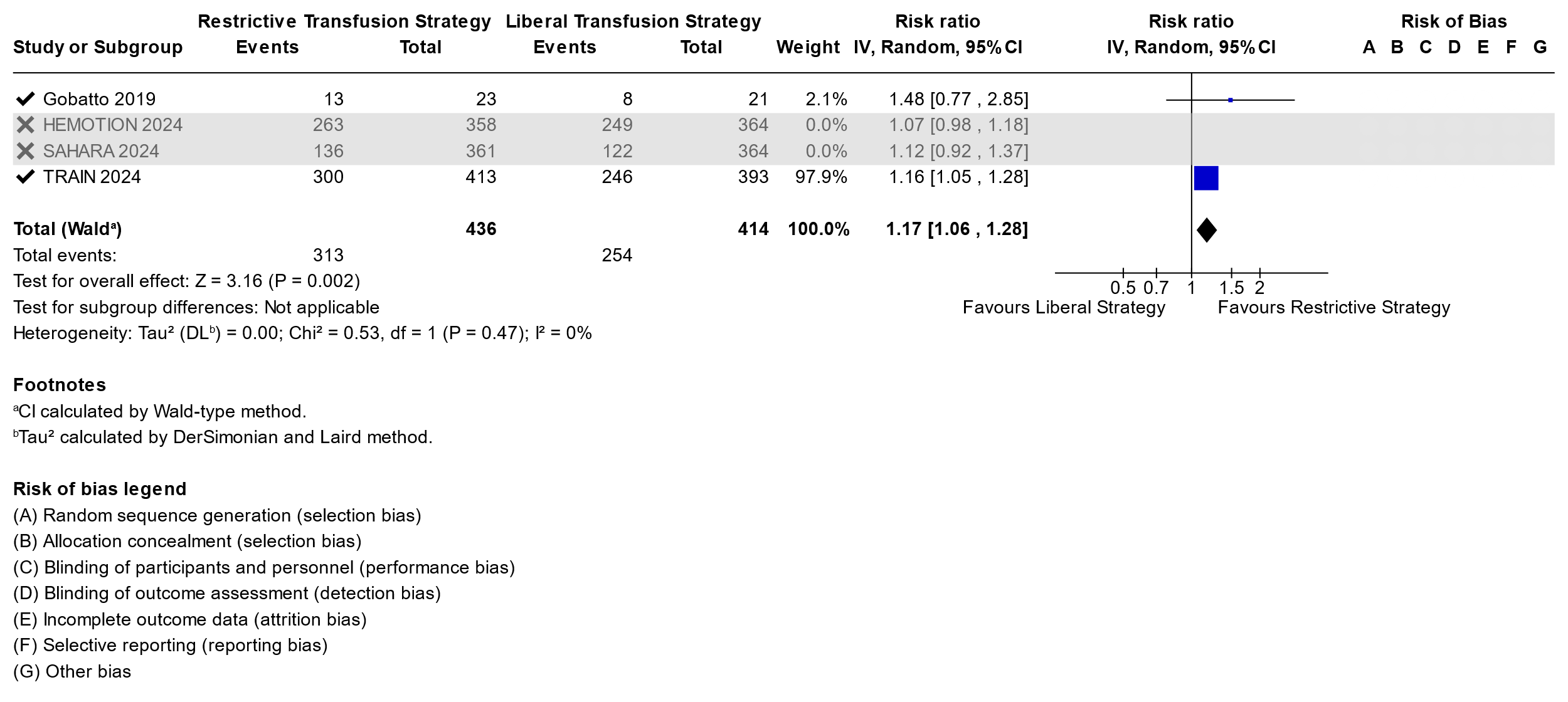


Figure 22 illustrates a higher incidence of UNOs at 6 months in patients who received the Restrictive Strategy compared to those under the Liberal Strategy, particularly when the Liberal transfusion threshold was set at Hb ≤ 9 g/dL. (A) When the Liberal Strategy was initiated at an Hb threshold of ≤ 10 g/dL, no substantial difference was observed between the two strategies (p = 0.06). In contrast, (B) a lower Hb threshold of ≤ 9 g/dL revealed a statistically significant difference between the transfusion strategies (p = 0.002).

**Figure 23. Impact of Transfusion Strategy on Sepsis or Septic shock Incidence, Stratified by Liberal Transfusion Threshold (Hb ≤ 9 g/dL vs. Hb ≤ 10 g/dL)**

**Sepsis or Septic shock**

**A.
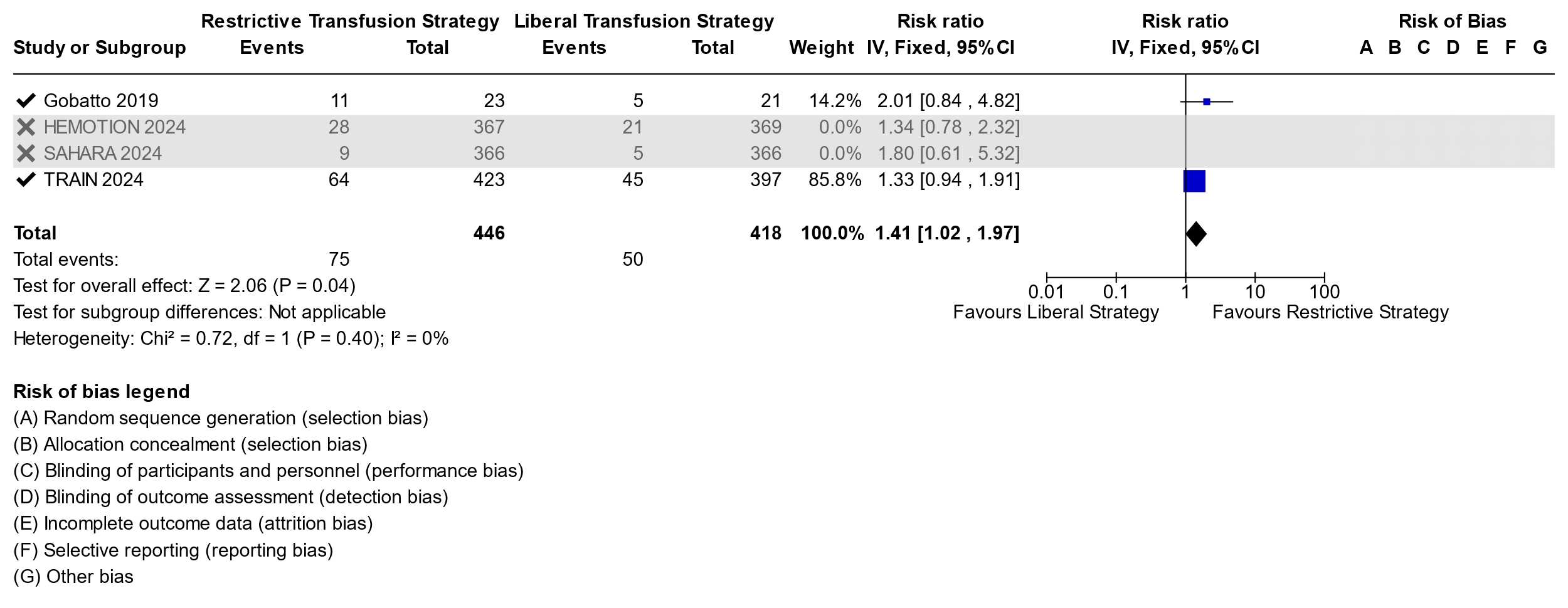
B.**
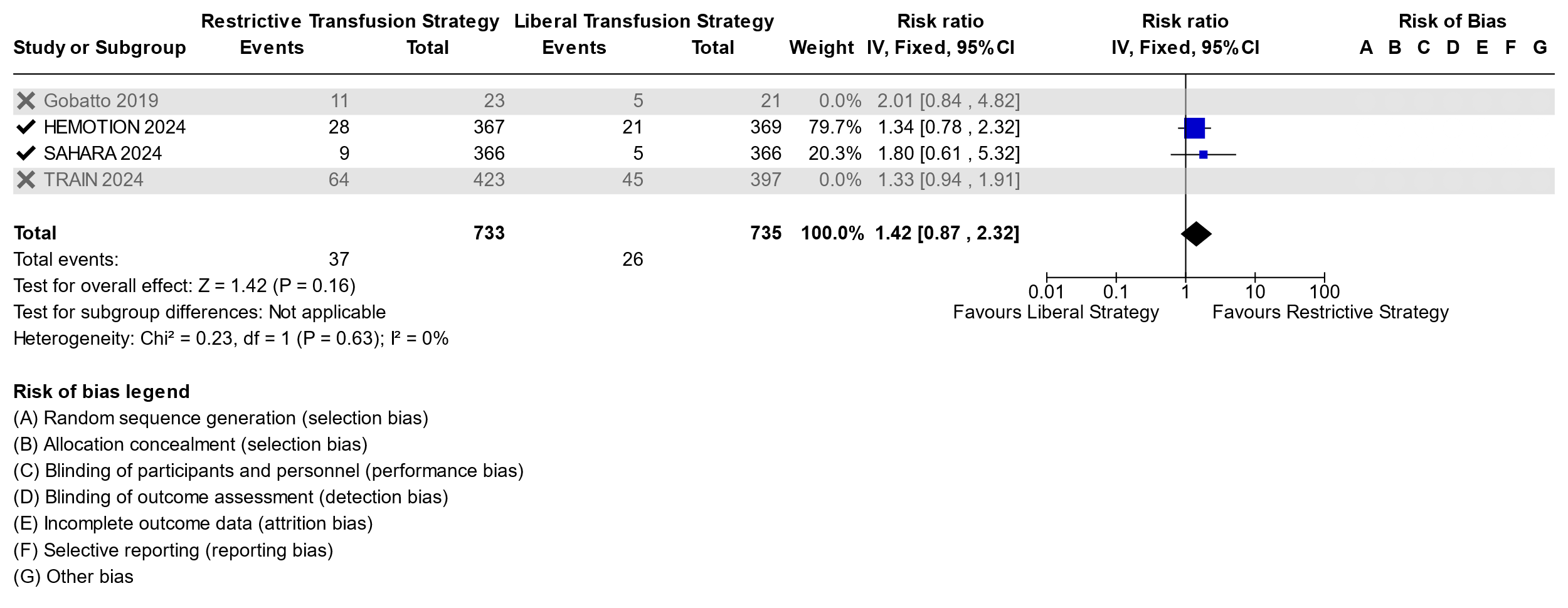


Figure 23 illustrates a higher incidence of sepsis or septic shock in patients who received the Restrictive Strategy compared to those under the Liberal Strategy, particularly when the Liberal transfusion threshold was set at Hb ≤ 9 g/dL. (A) When the Liberal Strategy was initiated at an Hb threshold of ≤ 10 g/dL, no significant difference was observed between the two strategies (p = 0.16). In contrast, (B) a lower Hb threshold of ≤ 9 g/dL revealed a statistically significant difference between the transfusion strategies (p = 0.04).

Figure 24. A Meta-regression Analysis


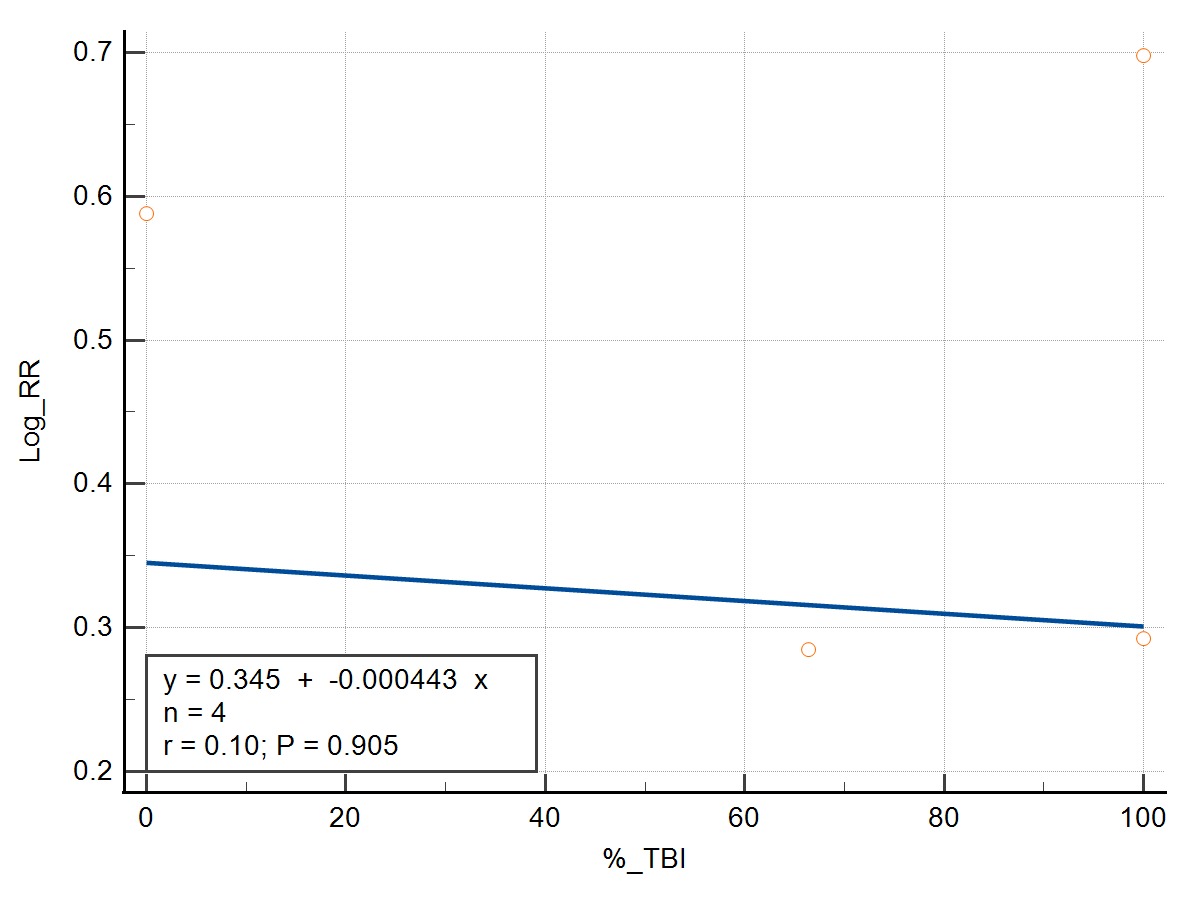


Figure 24 shows the relationship between variations in ABI types and sepsis composite outcomes, indicating no substantial association between the two, as p > 0.05.
