# Supplementary material for: Liberal transfusion strategies reduce sepsis risk and improve neurological recovery in acute brain injury: an updated systematic review and meta-analysis
Source: Crit Care. 2025 May 6;29:181. doi: 10.1186/s13054-025-05397-5 (PMC12057087; doi:10.1186/s13054-025-05397-5)
Supplement: Supplementary file 2 — Additional file 2. [file 13054_2025_5397_MOESM2_ESM.docx]

**Term definitions:**

**Sepsis**: Defined as infection accompanied by organ dysfunction, hypoperfusion, or hypotension. Confirmed by infection and at least two SIRS criteria (e.g., fever, hypothermia, leukocytosis), along with organ dysfunction (e.g., hypoxemia, elevated creatinine, coagulopathy). Indicators include hyperlactatemia and prolonged capillary refill.

**Septic Shock**: Sepsis with persistent hypotension despite adequate fluid resuscitation and perfusion abnormalities, such as lactic acidosis, oliguria, or acute mental status changes.

**Acute Respiratory Distress Syndrome (ARDS)**:

ARDS must meet all the following criteria:

- Acute onset
- PaO2/FiO2 ≤300
- Bilateral infiltrates on imaging
- No clinical evidence of left atrial hypertension.

Severity is classified as:

- Mild: 200 < PaO2/FiO2 ≤ 300
- Moderate: 100 < PaO2/FiO2 ≤ 200
- Severe: PaO2/FiO2 ≤ 100

**Unfavorable Neurological Outcomes**:

Defined as a Modified Rankin Scale (mRS) score ≥4. The mRS is a measure of functional independence, where:

- **4**: Moderately severe disability, requiring assistance for walking and attending to bodily needs.
- **5**: Severe disability, bedridden, incontinent, and requiring constant care.
- **6**: Death​

Unfavorable neurological outcomes are often evaluated using scales like the Glasgow Outcome Scale-Extended (GOS-E), which measures neurological recovery and functional outcomes after a brain injury, where:

- **1**: Dead
- **2**: Vegetative State: Unresponsive and speechless.
- **3**: Lower Severe Disability: Conscious but dependent for daily life; requires frequent assistance at home.
- **4**: Upper Severe Disability: Can be left alone for more than 8 hours but unable to travel or shop without assistance.
- **5**: Lower Moderate Disability: Unable to work or can only work in a sheltered environment.

**Table 2. Risk of bias summary for randomized studies (RoB 2)**

| **Study** | **Bias from randomization process** | **Bias due to deviations from intended interventions** | **Bias due to missing outcome data** | **Bias in measurement of the outcomes** | **Bias in selection of the reported result** | **Overall risk of bias** |
| --- | --- | --- | --- | --- | --- | --- |
| HEMOTION | Low | Low | Low | Low | Low | Low |
| TRAIN | Low | Low | Low | Low | Low | Low |
| SAHARA | Low | Some concerns | Low | Low | Low | Some concerns |
| Gobatto 2019 | Low | Some concerns | Low | Low | Low | Some concerns |
| McIntyre 2006 | Low | Low | Low | Some concerns | Low | Some concerns |

Table 2 summarizes the risk of bias assessments for the included studies. The majority of studies were determined to have a low-to-some-concern overall risk of bias, with no studies classified as being at high risk.

**Pooled analysis of all included studies**

**Figure 4. Acute Respiratory Distress Syndrome (ARDS) risk:**

**Restrictive Transfusion Strategy (RTS) versus Liberal Transfusion Strategy (LTS).**


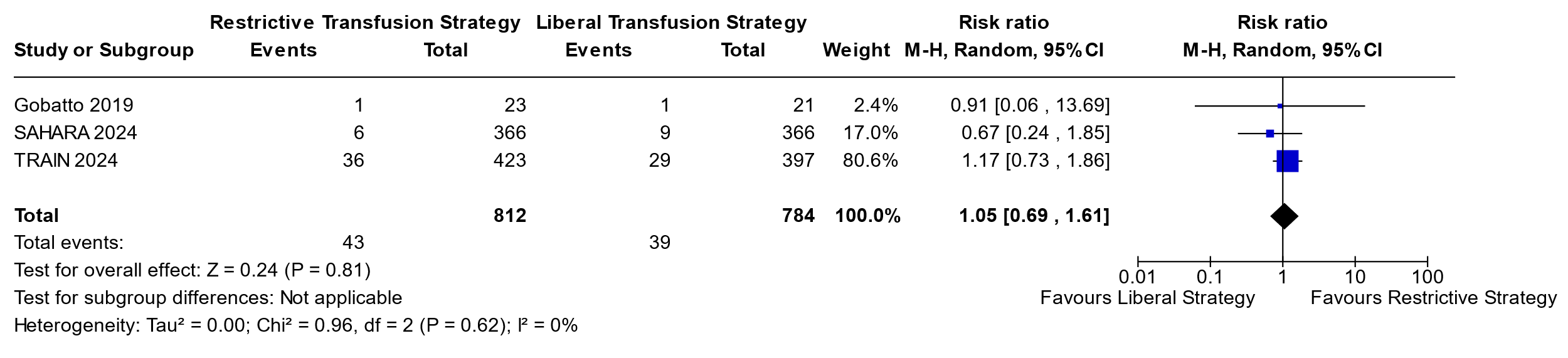


Figure 4. There were no noticeable differences in ARDS risk for patients treated with RTS, compared to LTS therapy (RR 1.05; 95% CI 0.69-1.61; p=0.81).

**Figure 5. Venous thromboembolism risk:**

**Restrictive Transfusion Strategy (RTS) versus Liberal Transfusion Strategy (LTS).**


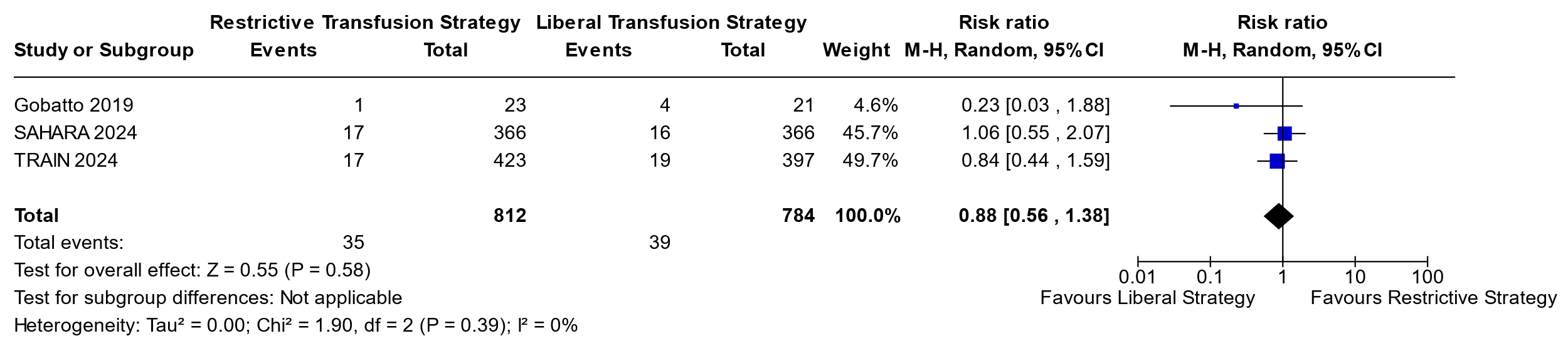


Figure 5. There were no noticeable differences in venous thromboembolism risk for patients treated with RTS, compared to LTS therapy (RR 0.88; 95% CI 0.56-1.38; p=0.58).

**Figure 7. In-hospital mortality:**

**Restrictive Transfusion Strategy (RTS) versus Liberal Transfusion Strategy (LTS).**


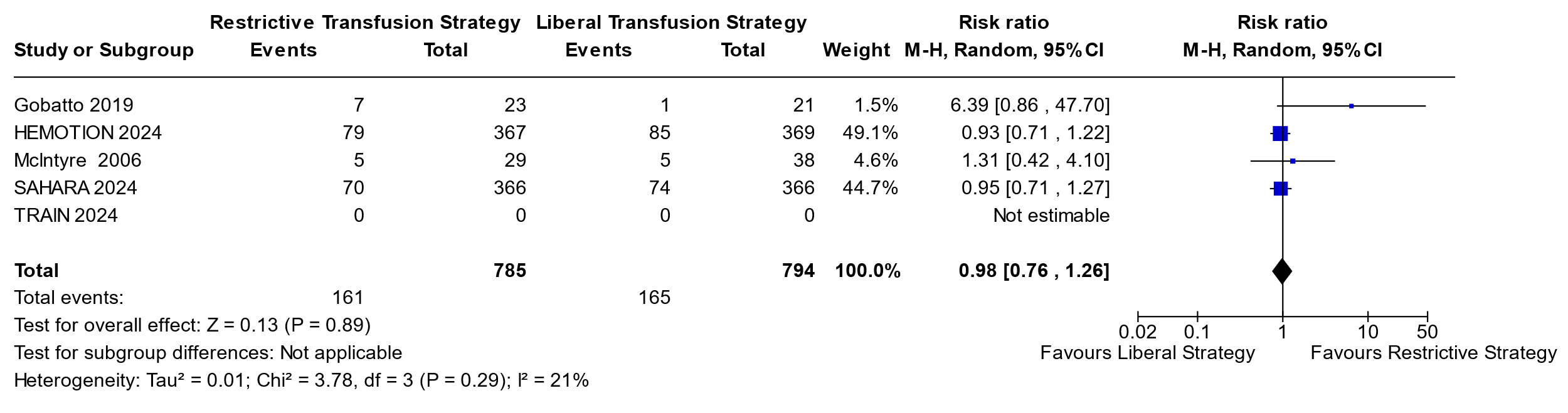


Figure 7. There were no noticeable differences in In-hospital mortality for patients treated with RTS, compared to LTS therapy (RR 0.98; 95% CI 0.76-1.26; p=0.89)

**Rationale thoughts for exclusion of Robertson 2014 trial, forest plots.**

**Figure 8. ROBERTSON 2014 Inclusion, and exclusion for UNOs at 6 months:**


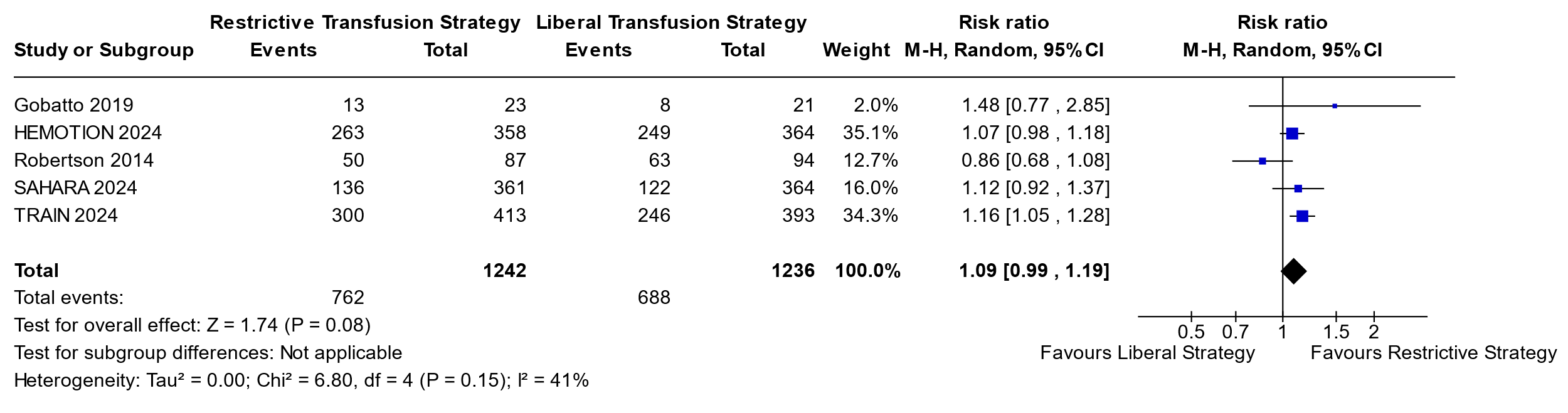


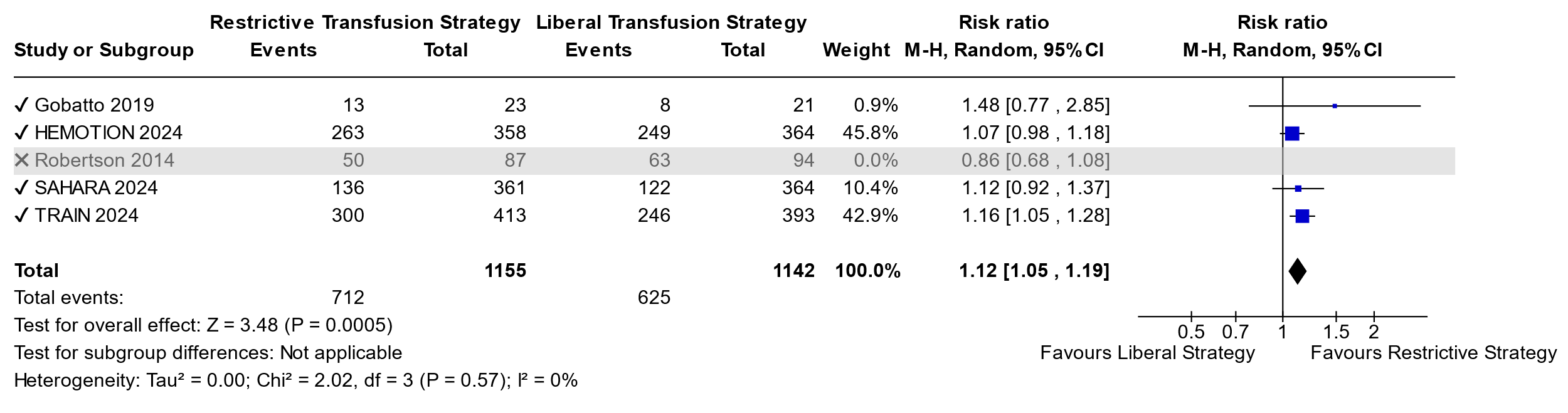


Figure 8 present the impact of including and excluding the ROBERTSON 2014 trial on the meta-analysis of UNOs at 6 months. The upper forest plot demonstrates the inclusion of the ROBERTSON 2014 trial, resulting in an insignificant p-value (p = 0.08) and a 95% confidence interval (0.99; 1.19) that includes the null value (1). Conversely, the lower forest plot shows the exclusion of the trial, yielding a statistically significant result (p = 0.0005), with a 95% confidence interval that excludes the null value.

**Figure 10. ROBERTSON 2014-Best Case Scenario for UNOs at 6 months. All missing data are not counted in the events group**.


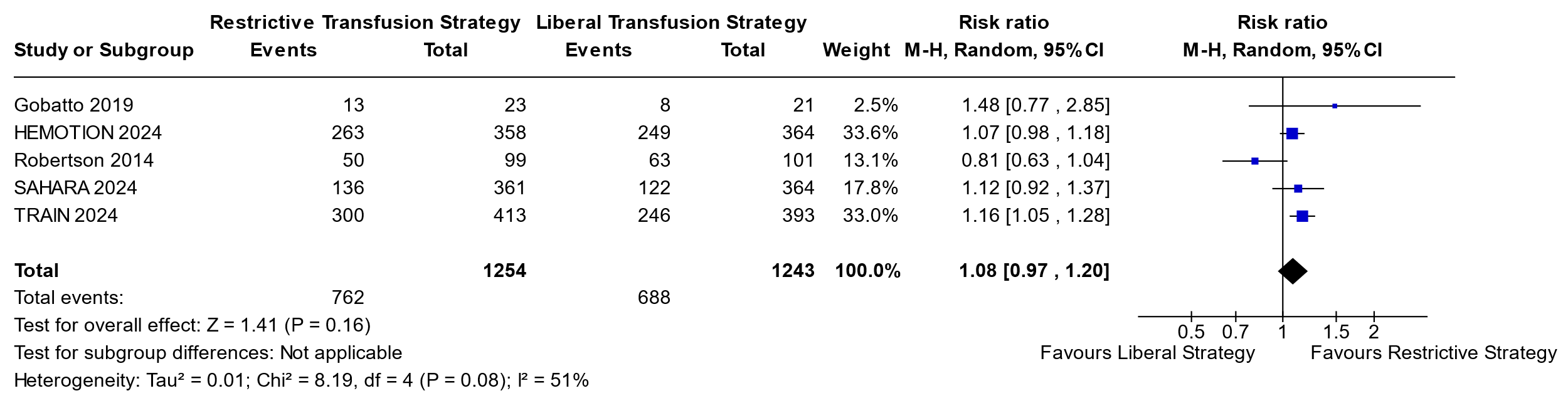


**Figure 9. ROBERTSON 2014-Worst Case Scenario for UNOs at 6 months. All missing data are counted in the events group**.


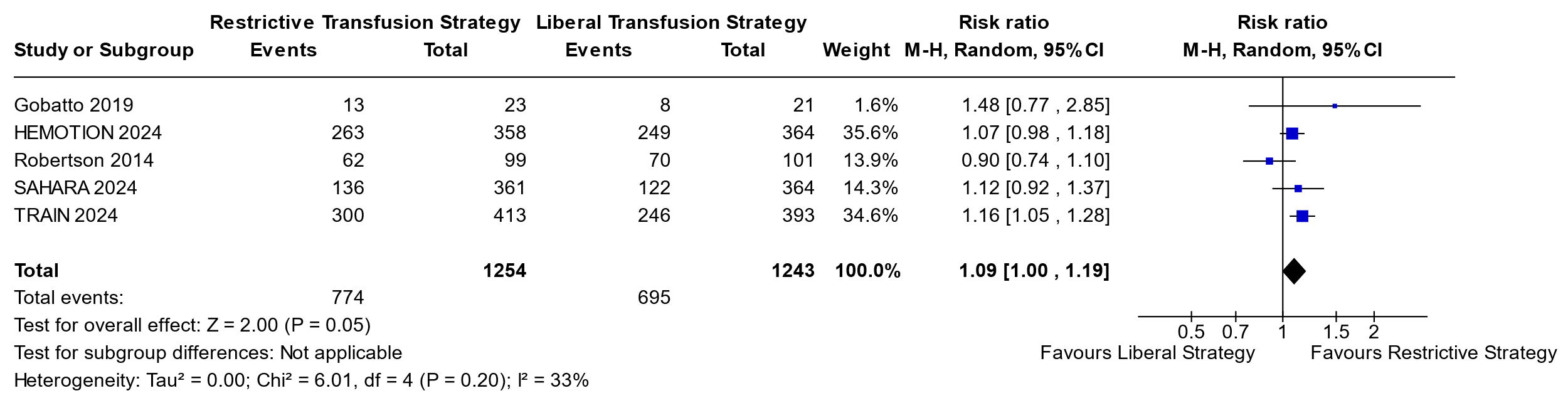


Figures 9-10. Best- and worst-case scenarios for the ROBERTSON 2014 trial on UNOs 6-month outcomes. The upper forest plot (Figure 10) represents the best-case scenario for the ROBERTSON 2014 trial, showing a statistically insignificant result (p = 0.16) with a 95% confidence interval (CI) of 0.97–1.20, which includes the null value (1). The lower forest plot (Figure 9) depicts the worst-case scenario for the same trial, demonstrating a borderline statistical result (p = 0.05) with a 95% CI of 1.00–1.19, also including the null value. These findings suggest a potential risk of bias associated with the ROBERTSON 2014 trial, influencing the interpretation of its impact on UNOs 6-month outcomes.

**Figure 11. ROBERTSON 2014 Inclusion, and exclusion for Sepsis or Septic shock risk:**


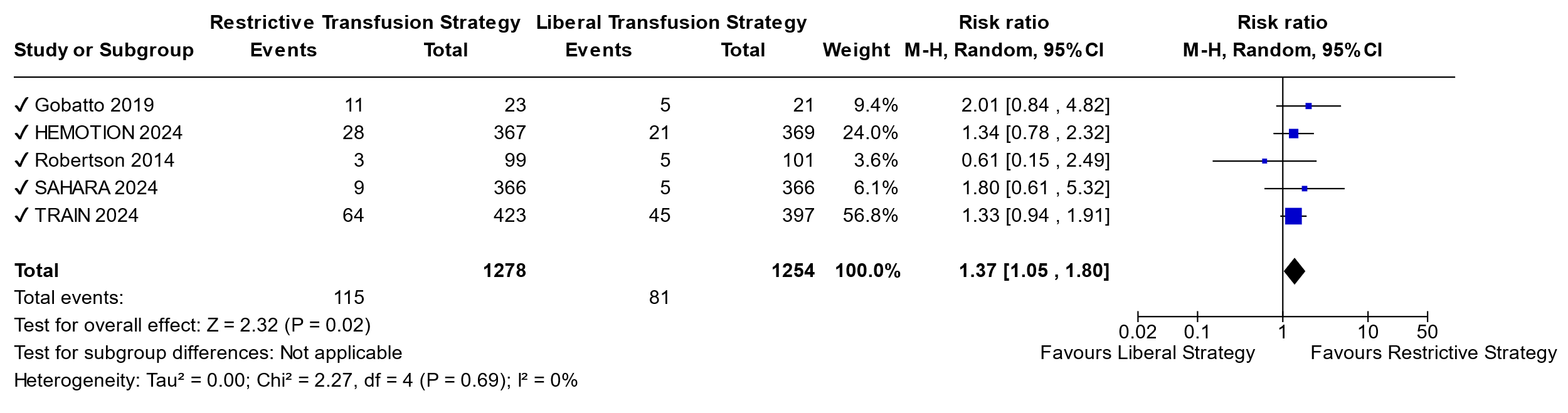


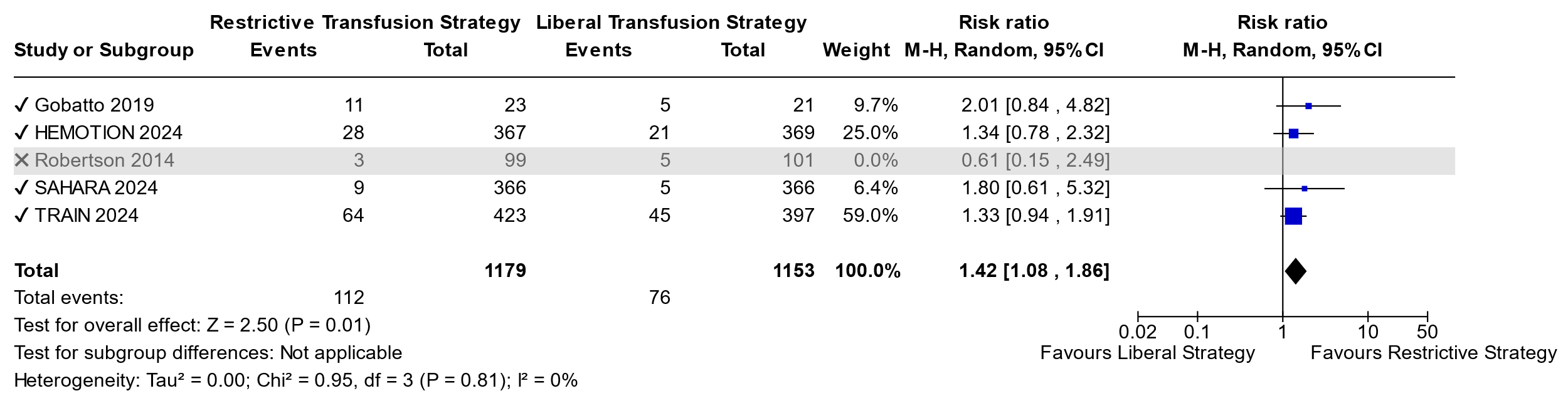


Figure 11. Impact of including and excluding the ROBERTSON 2014 trial on the meta-analysis of Sepsis or Septic Shock risk. The upper forest plot shows the inclusion of the ROBERTSON 2014 trial, yielding a statistically significant result (p = 0.02) with a 95% confidence interval (CI) of 1.05–1.80, which excludes the null value (1). The lower forest plot illustrates the exclusion of the ROBERTSON 2014 trial, maintaining statistical significance (p = 0.01) with a 95% CI of 1.08–1.86, also excluding the null value. These results indicate a consistent association between the intervention and the increased risk of Sepsis or Septic Shock, irrespective of the inclusion of the ROBERTSON 2014 trial.

**Subgroup analysis of 2024 RCTs, forest plots**

**Figure 12. Sepsis or Septic shock risk.**


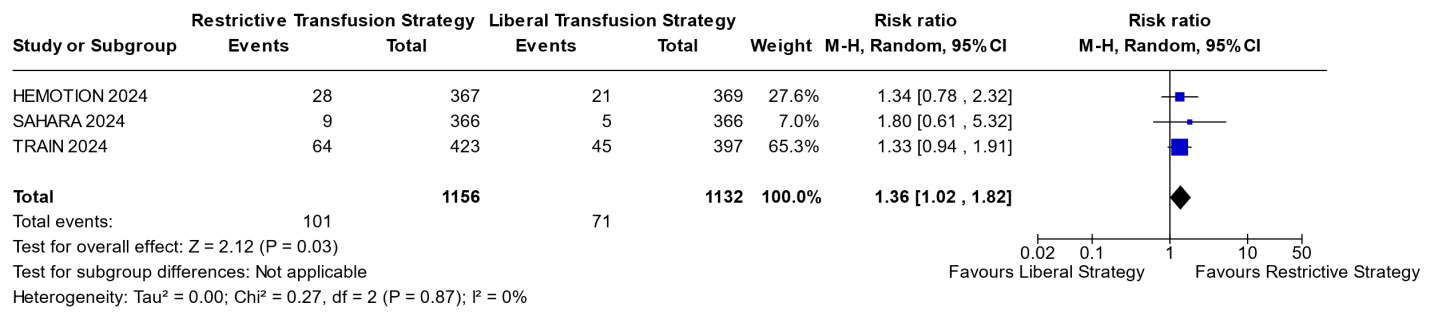


Figure 12 illustrates the comparison of sepsis or septic shock outcomes between RTS and LTS in RCTs 2024 subgroup. The analysis reveals a statistically significant difference (p = 0.03), with a risk ratio greater than 1 and a 95% confidence interval (1.02; 1.82) that excludes the null value of 1. This indicates a higher risk associated with RTS compared to LTS.

**Figure 13. UNOs at 6-month risk.**


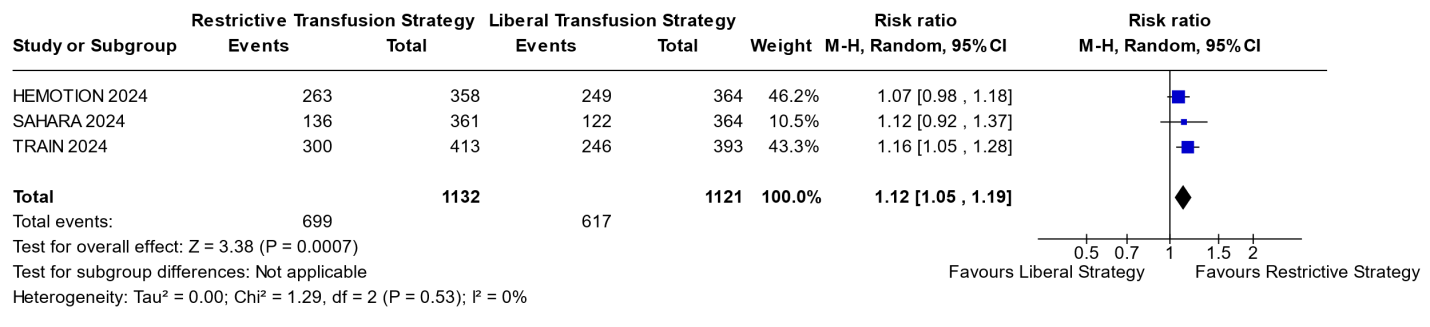


Figure 13 illustrates the comparison of UNOs at 6-month outcomes between RTS and LTS in the RCTs 2024 subgroup. The analysis reveals a statistically noticeable difference (p = 0.0007), with a risk ratio greater than 1 and a 95% confidence interval (1.05; 1.19) that excludes the null value of 1. This indicates a higher risk associated with RTS compared to LTS.

**Figure 14. Mortality at ICU.**

**
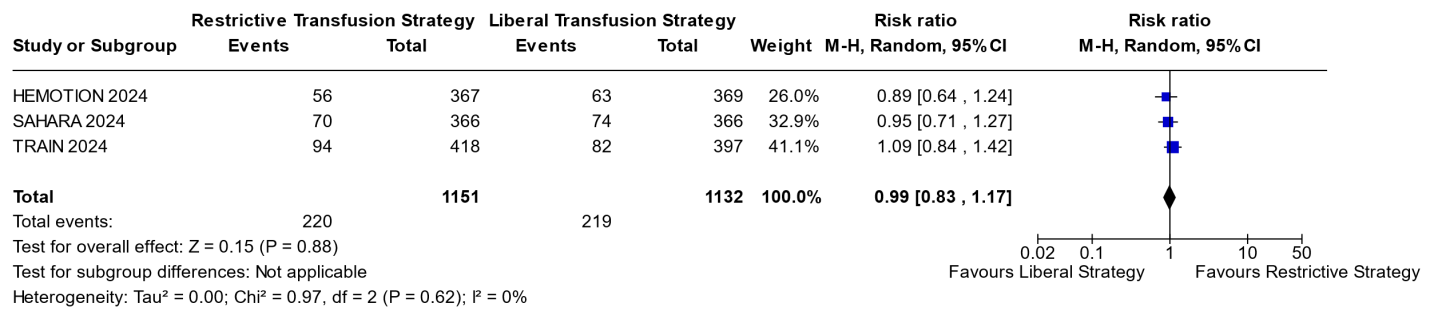
**

Figure 14 illustrates the comparison of mortality risk in the ICU between RTS and LTS in RCTs 2024 subgroup. The analysis reveals no significant difference (p = 0.88), with a risk ratio of 0.99 and a 95% confidence interval (0.83; 1.17) that includes the null value of 1. These findings indicate no discernible difference in mortality risk between the two strategies.

**Figure 15. Funnel plot for mortality in ICU outcome**


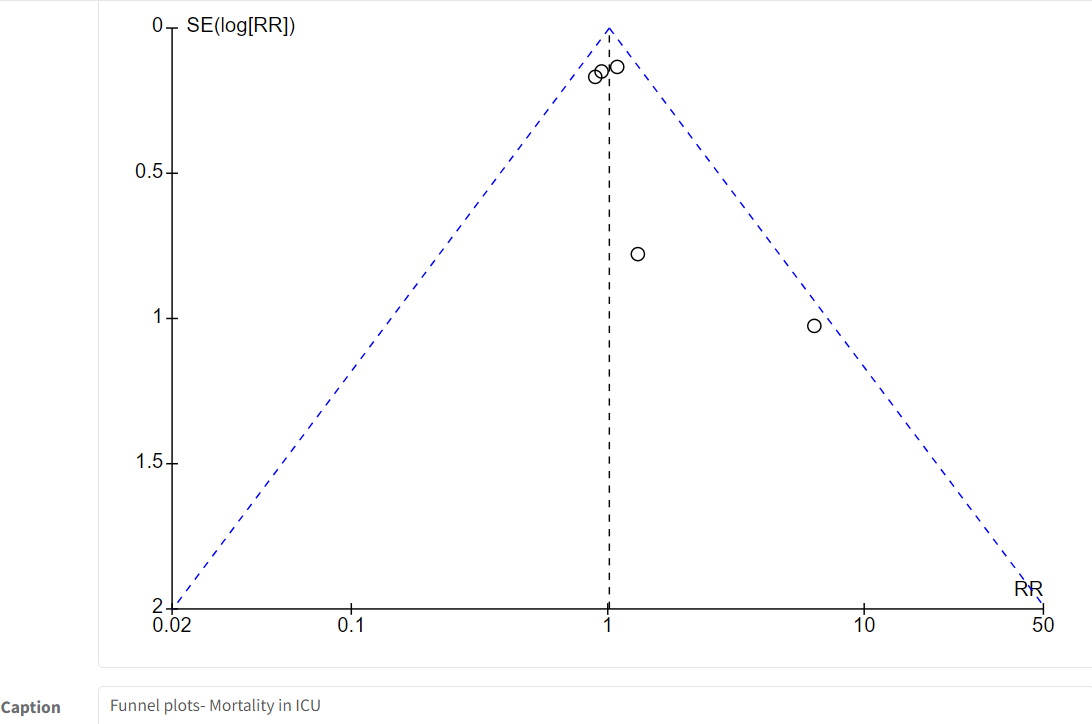


Figure 15 shows funnel plot for mortality in ICU outcome. The plot illustrates that most large-scale studies are symmetrically distributed near the top of the funnel, indicating minimal publication bias among these studies. In contrast, smaller-scale studies exhibit an asymmetrical distribution towards the base of the funnel, with a noticeable absence of studies on the left side of the base, suggesting potential publication bias or heterogeneity in these smaller studies.

**Subgroup analysis of UNOs at 6 months with either GOS or GOS-E, excluding SAHARA (mRS scale):**

**Figure 16. UNOs at 6 months.**

**
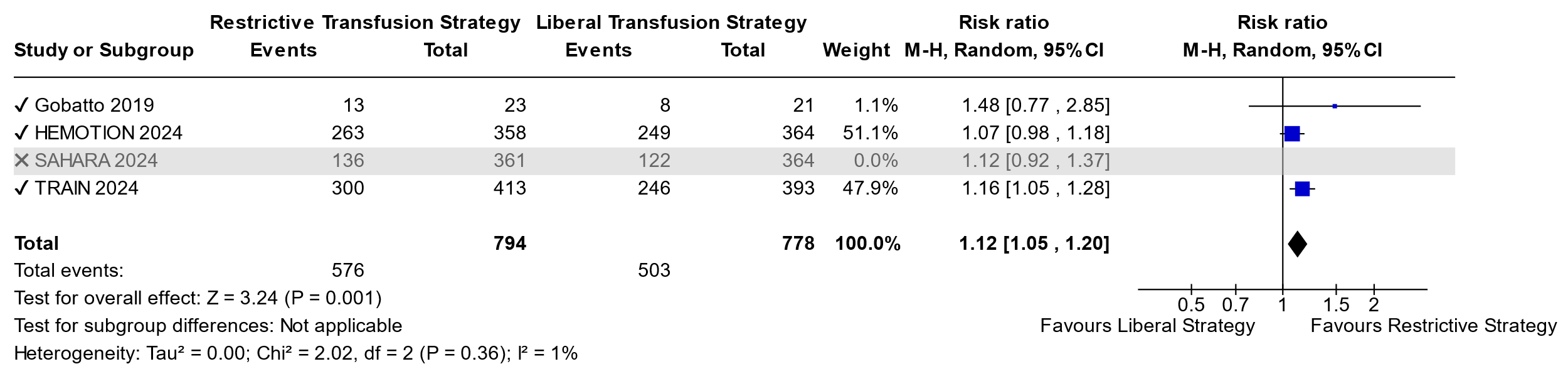
**

Figure 16 illustrates the comparison of UNOs at 6 months between RTS and LTS. The analysis reveals a statistically significant difference (p = 0.001), with a risk ratio of 1.12 and a 95% confidence interval (1.05; 1.20) that excludes the null value of 1. These findings indicate a discernible difference in UNOs at 6 months between the two strategies.

**Leave-one-out analysis of UNOs at 6 months, and Sepsis or Septic Shock Outcomes, exclusion of TRAIN:**

**Figure 17. Sepsis or Septic Shock risk.**

**
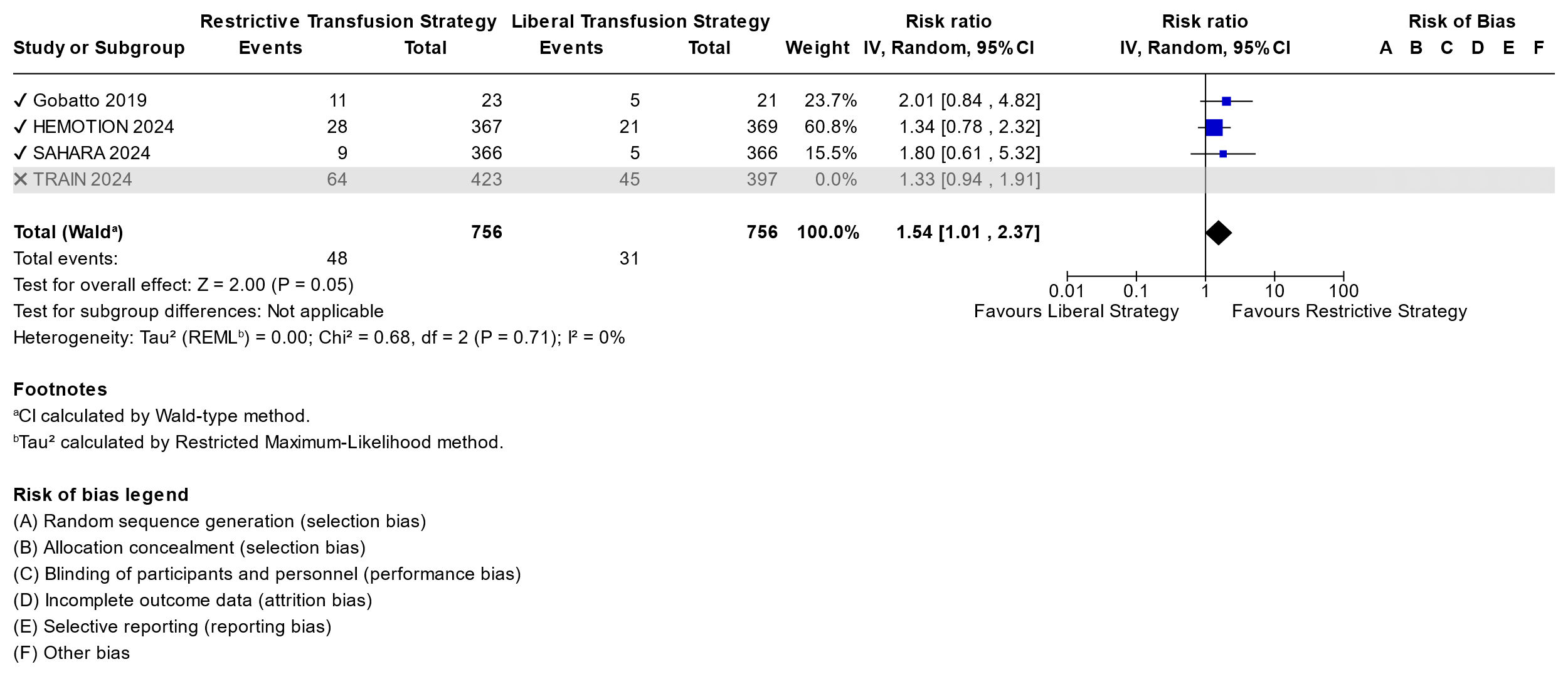
**

Figure 17 illustrates the comparison of Sepsis or Septic Shock risk between RTS and LTS. The analysis reveals a statistically significant difference (p = 0.05), with a risk ratio of 1.54 and a 95% confidence interval (1.01; 1.20) that excludes the null value of 1. These findings indicate a weak difference in Sepsis or Septic Shock risk between the two strategies.

**Figure 18. UNOs at 6 months,**

**
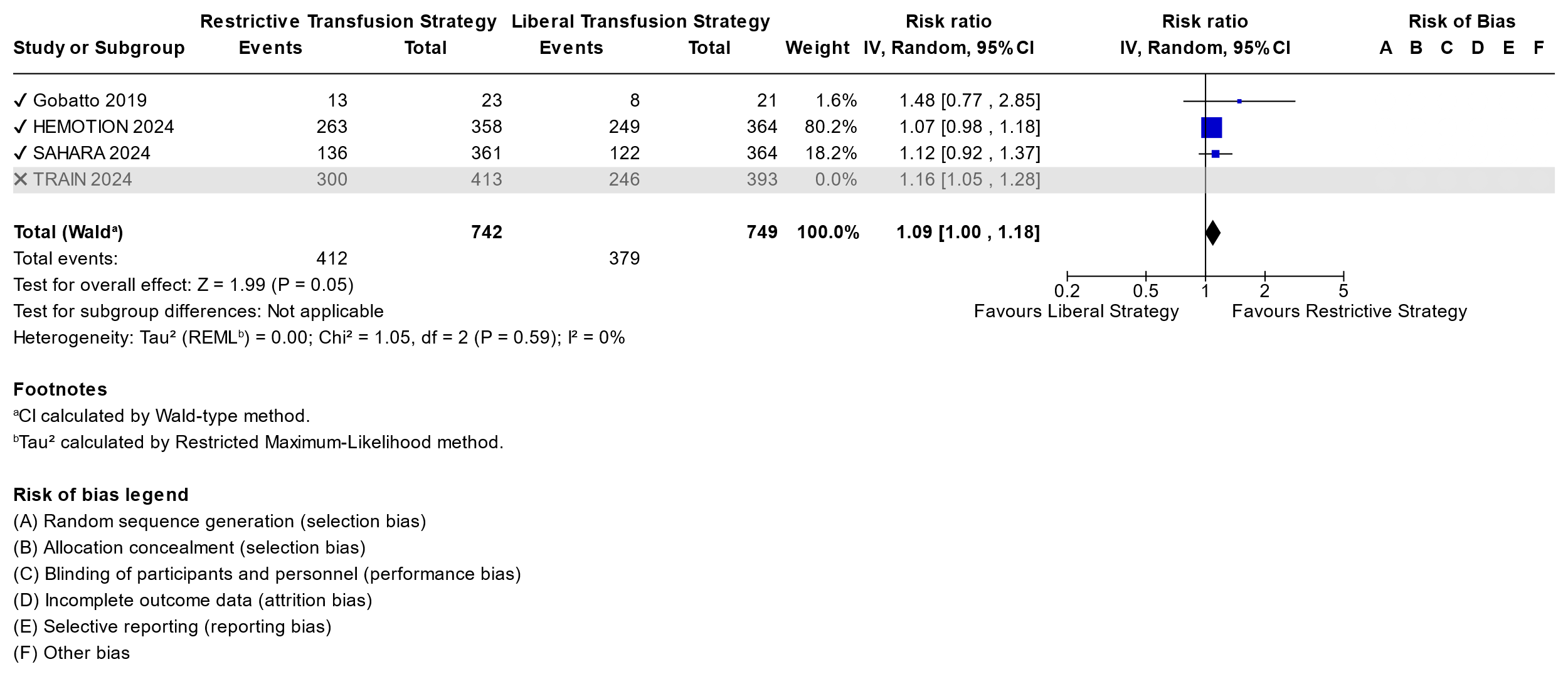
**

Figure 18 illustrates the comparison of UNOs at 6 months between RTS and LTS. The analysis reveals a statistically significant difference (p = 0.05), with a risk ratio of 1.09 and a 95% confidence interval (1.00; 1.18) that includes the null value of 1. These findings indicate a borderline difference in UNOs at 6 months between the two strategies.

**Table 3.Unfavorable Neurological Outcomes Defined By Individual Trials**

|  | **HEMOTION, 2024**  **(N=736)** | | **TRAIN, 2024**  **(N=820)** | | **SAHARA, 2024**  **(N=732)** | | **Gobatto, 2019**  **(N=44)** | | **McIntyre, 2006**  **(N=67)** | |
| --- | --- | --- | --- | --- | --- | --- | --- | --- | --- | --- |
| **Definitions** | LTS*  (n=369) | RTS*  (n=367) | LTS  (n=397) | RTS  (n=423) | LTS  (n=366) | RTS  (n=366) | LTS  (n=21) | RTS  (n=23) | LTS  (n=38) | RTS  (n=29) |
| UNOs^$^ scale,  at 6 months | ‘‘The primary outcome was centrally assessed **at 6 months** by trained personnel who were unaware of the group assignment. **The Glasgow Outcome Scale–Extended (GOS-E)** comprises eight ranking levels from 1 (death) to 8 (upper good recovery).  A sliding dichotomy was used to categorize scores as favorable or unfavorable according to each patient’s baseline prognosis. Analyses were adjusted for site (random intercept) and sex. The sliding dichotomy is based on the TBI-IMPACT prognostic model, which includes admission characteristics (age, GCS motor score, pupil reactivity, status with regard to hypoxemia and hypotension, injury classification on the basis of CT, the presence or absence of traumatic subarachnoid hemorrhage on CT scan and of epidural hematoma, and blood glucose and hemoglobin levels). When necessary, conditional estimation was used for missing covariates to calculate an individual TBI-IMPACT score for each patient.  Patients were divided into thirds according to their predicted risk of an unfavorable outcome: patients in the **worst prognosis group** were considered to have an **unfavorable outcome if the GOS-E score at 6 months was 3 or lower** (i.e., death, vegetative state, or lower severe disability); patients in **the intermediate prognosis** group were considered to have **an unfavorable outcome if the GOS-E score was 4 or lower** (i.e., death, vegetative state, lower severe disability, or upper severe disability); and patients in **the best prognosis group** were considered to have **an unfavorable outcome if the GOS-E score was 5 or lower** (i.e., death, vegetative state, lower severe disability, upper severe disability, or lower moderate disability)’’ -HEMOTION^13^ | | ‘‘Neurological outcome **at 180 days** after randomization was assessed using **the Glasgow Outcome Scale Extended (GOS-E),** dichotomized as **unfavorable (GOS-E score, 1-5)** or favorable (GOS-E score, 6-8); the score ranges from 1 to 8, with death included in the scale (GOS-E score, 1) and a higher score indicating a better outcome’’-TRAIN^8^ | | ‘‘For purposes of the primary outcome, an **unfavorable neurologic outcome** was defined as **a modified Rankin scale score of 4 or higher measured at 6 months** in a blinded fashion by an independent assessor.  Scores on the modified Rankin scale are as follows: 0, complete recovery; 1, no significant disability despite symptoms; 2, slight disability (unable to carry out previous activities but able to look after own affairs); 3, moderate disability (help needed but walks without assistance); 4, moderately severe disability (unable to walk unassisted or to address own bodily needs); 5, severe disability (incontinence and total nursing care); and 6, dead’’- SAHARA^14^ | | ‘‘Neurological status was evaluated using **the Glasgow Outcome Scale (GOS)** at hospital discharge and **6 months** after hospital discharge. The neurological status evaluation was made by ambulatory consultation or home visit. When the patient was unable to come to an ambulatory consultation or a home visit was not possible, the neurological status evaluation was made by a telephone call to the patient or to a family member  **Dichotomous UNOs defined as GOS-3’’**  -Gobatto et al 2019^24^ | | N/A | |

**Table 4. Summary of Shared Con-founders Accounted for by Randomization in Each Trial**

| **Trial** | **Hemoglobin level at randomization** | **Any severity/ prognostic scores measured at admission** | **GCS at admission** | **Any prior red-cell transfusion** | **Medical history or Significant comorbidity reports** | **Pupil reactivity,**  **one or both eyes** | **Chronic**  **conditions**  **(anemia/ cancer/ heart failure/ COPD/ diabetes/ hypertension)** |
| --- | --- | --- | --- | --- | --- | --- | --- |
| HEMOTION | Yes (mean:9.1g/dl) | Yes  (Injury Severity Score) | Yes,  Only motor | Yes | Yes | Yes | Yes (heart failure, and anemia) |
| TRAIN | Yes  (median:8.5g/dl) | Yes  (APACHE II score) | Yes | No | Yes | Yes | Yes (diabetes, cancer, heart failure, and COPD) |
| SAHARA | Yes  (median:9.5g/dl) | Yes  (Modified Fisher scale, SAH) | Yes | Yes | Yes | No | Yes (anemia, heart failure, hypertension) |
| Gobatto 2019 | No | Yes  (Injury Severity Score, and SAPS 3) | Yes | Yes | No | Yes | No |
| McIntyre 2006 | No | Yes  (Injury Severity Score, and APACHE II score) | Yes | Yes | Yes | No | No |

Table 4 summarizes the potential con-founders addressed through randomization in each trial. The list is not exhaustive, and the specific con-founders vary across studies. While randomization helps mitigate confounding, differences in ICU management and randomization strategies may still influence the findings.

| **Section and Topic** | **Item #** | **Checklist item** | **Page where item is reported** |
| --- | --- | --- | --- |
| **TITLE** | | |  |
| Title | 1 | Identify the report as a systematic review. | 1 |
| **ABSTRACT** | | |  |
| Abstract | 2 | See the PRISMA 2020 for Abstracts checklist. | 2 |
| **INTRODUCTION** | | |  |
| Rationale | 3 | Describe the rationale for the review in the context of existing knowledge. | 4 |
| Objectives | 4 | Provide an explicit statement of the objective(s) or question(s) the review addresses. | 4 |
| **METHODS** | | |  |
| Eligibility criteria | 5 | Specify the inclusion and exclusion criteria for the review and how studies were grouped for the syntheses. | 6 |
| Information sources | 6 | Specify all databases, registers, websites, organisations, reference lists and other sources searched or consulted to identify studies. Specify the date when each source was last searched or consulted. | 6 |
| Search strategy | 7 | Present the full search strategies for all databases, registers and websites, including any filters and limits used. | 6 |
| Selection process | 8 | Specify the methods used to decide whether a study met the inclusion criteria of the review, including how many reviewers screened each record and each report retrieved, whether they worked independently, and if applicable, details of automation tools used in the process. | 7 |
| Data collection process | 9 | Specify the methods used to collect data from reports, including how many reviewers collected data from each report, whether they worked independently, any processes for obtaining or confirming data from study investigators, and if applicable, details of automation tools used in the process. | 7 |
| Data items | 10a | List and define all outcomes for which data were sought. Specify whether all results that were compatible with each outcome domain in each study were sought (e.g. for all measures, time points, analyses), and if not, the methods used to decide which results to collect. | 7 |
|  | 10b | List and define all other variables for which data were sought (e.g. participant and intervention characteristics, funding sources). Describe any assumptions made about any missing or unclear information. | N/A |
| Study risk of bias assessment | 11 | Specify the methods used to assess risk of bias in the included studies, including details of the tool(s) used, how many reviewers assessed each study and whether they worked independently, and if applicable, details of automation tools used in the process. | 7 |
| Effect measures | 12 | Specify for each outcome the effect measure(s) (e.g. risk ratio, mean difference) used in the synthesis or presentation of results. | 7 |
| Synthesis methods | 13a | Describe the processes used to decide which studies were eligible for each synthesis (e.g. tabulating the study intervention characteristics and comparing against the planned groups for each synthesis (item #5)). | N/A |
|  | 13b | Describe any methods required to prepare the data for presentation or synthesis, such as handling of missing summary statistics, or data conversions. | N/A |
|  | 13c | Describe any methods used to tabulate or visually display results of individual studies and syntheses. | N/A |
|  | 13d | Describe any methods used to synthesize results and provide a rationale for the choice(s). If meta-analysis was performed, describe the model(s), method(s) to identify the presence and extent of statistical heterogeneity, and software package(s) used. | 8 |
|  | 13e | Describe any methods used to explore possible causes of heterogeneity among study results (e.g. subgroup analysis, meta-regression). | 8 |
|  | 13f | Describe any sensitivity analyses conducted to assess robustness of the synthesized results. | 8 |
| Reporting bias assessment | 14 | Describe any methods used to assess risk of bias due to missing results in a synthesis (arising from reporting biases). | 8 |
| Certainty assessment | 15 | Describe any methods used to assess certainty (or confidence) in the body of evidence for an outcome. | 8 |
| **RESULTS** | | |  |
| Study selection | 16a | Describe the results of the search and selection process, from the number of records identified in the search to the number of studies included in the review, ideally using a flow diagram. | 10 |
|  | 16b | Cite studies that might appear to meet the inclusion criteria, but which were excluded, and explain why they were excluded. | 10 |
| Study characteristics | 17 | Cite each included study and present its characteristics. | 12 |
| Risk of bias in studies | 18 | Present assessments of risk of bias for each included study. | 20 |
| Results of individual studies | 19 | For all outcomes, present, for each study: (a) summary statistics for each group (where appropriate) and (b) an effect estimate and its precision (e.g. confidence/credible interval), ideally using structured tables or plots. | 13-16 |
| Results of syntheses | 20a | For each synthesis, briefly summarise the characteristics and risk of bias among contributing studies. | N/A |
|  | 20b | Present results of all statistical syntheses conducted. If meta-analysis was done, present for each the summary estimate and its precision (e.g. confidence/credible interval) and measures of statistical heterogeneity. If comparing groups, describe the direction of the effect. | 13-16 |
|  | 20c | Present results of all investigations of possible causes of heterogeneity among study results. | 19 |
|  | 20d | Present results of all sensitivity analyses conducted to assess the robustness of the synthesized results. | 15 |
| Reporting biases | 21 | Present assessments of risk of bias due to missing results (arising from reporting biases) for each synthesis assessed. | 20 |
| Certainty of evidence | 22 | Present assessments of certainty (or confidence) in the body of evidence for each outcome assessed. | 20 |
| **DISCUSSION** | | |  |
| Discussion | 23a | Provide a general interpretation of the results in the context of other evidence. | 21 |
|  | 23b | Discuss any limitations of the evidence included in the review. | 24 |
|  | 23c | Discuss any limitations of the review processes used. | 24 |
|  | 23d | Discuss implications of the results for practice, policy, and future research. | 26 |
| **OTHER INFORMATION** | | |  |
| Registration and protocol | 24a | Provide registration information for the review, including register name and registration number, or state that the review was not registered. | 7 |
|  | 24b | Indicate where the review protocol can be accessed, or state that a protocol was not prepared. | N/A |
|  | 24c | Describe and explain any amendments to information provided at registration or in the protocol. | N/A |
| Support | 25 | Describe sources of financial or non-financial support for the review, and the role of the funders or sponsors in the review. | 27 |
| Competing interests | 26 | Declare any competing interests of review authors. | 1 |
| Availability of data, code and other materials | 27 | Report which of the following are publicly available and where they can be found: template data collection forms; data extracted from included studies; data used for all analyses; analytic code; any other materials used in the review. | 27 |

**Table 5. PRISMA checklist**

*From:*  Page MJ, McKenzie JE, Bossuyt PM, Boutron I, Hoffmann TC, Mulrow CD, et al. The PRISMA 2020 statement: an updated guideline for reporting systematic reviews. BMJ 2021;372:n71. doi: 10.1136/bmj.n71. This work is licensed under CC BY 4.0. To view a copy of this license, visit <https://creativecommons.org/licenses/by/4.0/>
